# Optimizing sowing date for enhanced heat stress tolerance in canola (*Brassica napus* L.): Investigating impacts on seed yield, oil content, and fatty acids composition

**DOI:** 10.1016/j.heliyon.2025.e42138

**Published:** 2025-01-21

**Authors:** Seyed Ahmad Kalantar Ahmadi, Mohsen Sarhangi

**Affiliations:** Department of Agronomy and Horticultural Science, Safiabad Agricultural and Natural Resources Research and Education Center, Agricultural Research, Education and Extension Organization (AREEO), PO Box 333, Dezful, Iran

**Keywords:** Canola, Genotype, Heat stress, Oleic acid, Seed filling

## Abstract

Environmental conditions, including temperature and the occurrence of phenological stages at the optimum temperature, are effective factors on seed yield, oil content and fatty acids. An experiment was carried out as strip block based on randomized completed block design with three replications in Safiabad Agricultural and Natural Resources Research and Education Center of Dezful during two cropping seasons (2017–2019). Vertical factors consisted of six levels of sowing date (23 Sep, Oct 7, 22 Oct, Nov 6, 21 Nov, Dec 6), and horizontal factors were seven genotypes (Agamx, Hyola4815, Hyola50, Hyola401, Safi6, Zabol9 and Zabol13). Elevated temperatures resulting from postponed sowing dates during the silique formation and seed filling phases led to a decline in seed yield, oil content, and modifications in the fatty acid composition of the studied canola genotypes. The highest (43.04 %) and lowest (38.81 %) oil content over the two years of testing were attributed to the genotypes Hyola50 and Hyola4815, respectively. Postponing the sowing date contributed to a higher accumulation of oleic acid in the examined genotypes. The maximum oleic acid content (62.14 %) was observed on Dec. 6 for the genotype Hyola50, while the minimum oleic acid content (50 %) was recorded on Sep. 23 for the genotype Zabol9 during two years of the experiment. Variations in climatic conditions across the two experimental years elicited distinct responses in the studied genotypes based on the sowing date. In the first year, Agamax genotype produced the highest seed yield (3357 kg ha^−1^) on Oct 7th, but in the second year the highest seed yield (2888.9 kg ha^−1^) belonged to the second sowing date (Oct. 7) and Hyola50 genotype. Based on the test results, the susceptibility of canola genotypes to temperature, rainfall, and lodging during seed filling period varied between the two years of the experiment. The percentage reduction in seed yield for the Hyola50 and Agamax genotypes was 32 % and 40 %, respectively. Climatic factors, particularly temperature and the synchronization of phenological stages with optimal thermal conditions, play a crucial role in determining seed yield, oil content, and fatty acid composition. Furthermore, the selection of heat-tolerant genotypes is essential for maintaining yield stability.

## 1 Introduction

Climate change resulting from global warming is a worldwide concern [1], and the increase in air temperature has led to reduced yield of many crops [2]. It is predicted that by 2050, the average global air temperature will increase by 2 °C negatively impacting agricultural production and potentially exposing around 50 million people to the risk of hunger [1].

Canola ranks among the most significant oilseed crops worldwide, and rising temperatures impact its developmental stages, seed yield, and oil composition [3]. The timing of sowing is vital for canola cultivation, as the flowering, siliquing, and seed-filling phases should ideally avoid overlap with late-season heat stress [4,5]. Optimal sowing date and early flowering ensure that flower and silique initiation stages align with favorable environmental conditions (temperature, radiation, moisture), promoting the fertility and development of silique-forming cells. Conversely, exposure of reproductive stages to heat at the end of the season and hot dry winds leads to reduced silique formation and seed yield [4]. Therefore, selecting an appropriate sowing date based on local climatic characteristics is crucial for achieving maximum canola seed yield [5,6].

Choosing the appropriate sowing date in each region should ensure optimal environmental conditions throughout the growing season for plants without exposure to environmental stresses [4,7,8]. Therefore, it is necessary to evaluate the response of genotypes to environmental conditions [9] and identifying the reaction of genotypes to changes in sowing dates and determining high-yielding genotypes can have a significant impact on expanding canola cultivation [10]. It has been reported that the quantity and quality of canola genotypes in autumn sowing (October) are higher compared to winter planting (January) [11]. Research results in rapeseed have shown that the number of flowers transformed into siliques is a determining factor for seed yield [3,12] and the production of assimilates during the flowering period determines the number of seeds per silique, while seed weight depends on the continuity of photosynthesis and assimilate production during the post-flowering period until maturity [12,13]. Considering that rapeseed reproductive organs are negatively affected by temperatures above 32 °C, observing the sowing date plays a crucial role in silique development and seed setting [3].

The temperature during the seed-filling phase affects both the quantity of oil and the composition of fatty acids, with higher temperatures during this time resulting in reduced seed oil content [3]. Environmental stresses, particularly elevated temperatures during seed filling, hinder the production of substrates necessary for triacylglycerol synthesis [3] and diminish fatty acid biosynthesis activities, leading to lower oil content in seeds [14]. An increase in temperature from 19 to 24 C° causes changes in canola fatty acids content [15], with the impact of high temperatures between flowering and seed filling stages on reducing seed oil content and fatty acid changes being more pronounced in sensitive canola genotypes [16]. The role of gibberellin signaling in activating genes involved in fatty acid metabolism and oxalate glycolysis pathway has also been reported [16].

At a certain stage of development, canola seeds contain chlorophyll and feature a thylakoid structure. Light exposure promotes fatty acid biosynthesis in green canola seeds, which may facilitate the production of cofactors and reductants [17,18]. The transcription factor BnWRI1 regulates the pathways of photosynthesis and fatty acid biosynthesis throughout seed development [19]. The lower chlorophyll *a*/b ratio in seeds compared to leaves enhances carbon efficiency in oil production [17]. Sucrose is transported from leaves to support seed growth and the synthesis of storage products; however, under high-temperature conditions, oil storage declines, and external sucrose fails to alleviate the adverse effects of heat stress on oil accumulation. Consequently, the metabolic pathway converting carbohydrates to triacylglycerols is disrupted. Additionally, heat stress conditions lead to decreased expression of certain genes associated with sugar transport in seeds [20]. Photosynthesis in canola embryos significantly boosts oil accumulation efficiency [17,21], but the activity of photosystem II (PSII) is inhibited by photosynthetic inhibitors, which considerably hampers biomass and oil production in embryos grown under fluorescent light [18]. Ultimately, damage to PSII and suppression of BnWRI1 gene activity are likely key factors contributing to reduced canola oil content under heat stress conditions [20].

Canola is one of the most important oilseeds cultivated in many parts of the world, and attention to agricultural factors such as appropriate sowing date and selecting adapted genotypes plays a crucial role in increasing the quantity and quality of this plant's oil. Many regions in Iran, including warm and dry areas, have faced increased temperatures due to climate change in recent years, posing challenges for farmers on how to escape terminal heat stress as well as choose tolerant and adapted genotypes. Since identifying and planting high-yielding genotypes compatible with climatic conditions can improve both the yield and quality of canola production, the present research aims to identify and introduce the best genotype for different planting dates.

## 2 Materials and methods

### 2.1 Experimental site

**Fig. 1:**
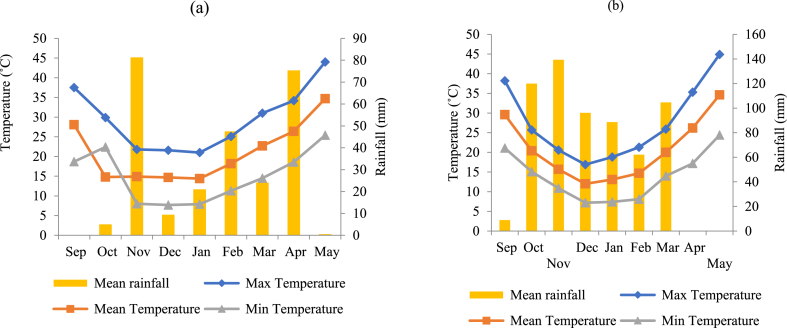
Mountly temperature (mean, maximum and minimum) and rainfall over the growing season 2017–2018 (a) and 2018–2019 (b) of canola in Dezful, Iran.

The current field experiment was conducted at the Safiabad Agricultural and Natural Resources Research and Education Center, situated at a latitude of 32°22′ N, longitude of 48°32′ E, and an elevation of 82 m above sea level in Khuzestan province, southwestern Iran. Fig. 1a and b illustrates the temperature and precipitation during the experimental period. The soil characteristics are detailed in Table 1.

**Table 1:** Characteristics of physical and chemical properties of soil used in the experiment.

Year	Soil texture	OC (%)	P (ppm)	K (ppm)	pH	EC (ds/m)	Zn (ppm)	Mn (ppm)	B (ppm)
2017–2018	Clay-Loam	0.62	8.7	181	7.74	0.77	0.74	4.8	0.65
2018–2019	Clay-Loam	0.67	10.2	186	7.68	0.86	0.78	4.6	0.67

OC: Organic carbon, P: Phosphorus, K: Potassium, pH: Potential of hydrogen, EC: Electrical conductivity, Zn: Zinc, Mn: Manganese, B: Boron.

This study was designed as strip plots within a randomized complete block design, featuring three replications over two cropping seasons (2017–2019) at the same research center. Seven canola cultivars, both domestic and international (Agamax, Hyola4815, Hyola50, Hyola401, Safi6, Zabol9, and Zabol13), were assessed across six different sowing dates (September 23, October 7, October 22, November 6, November 21, and December 6). The traits of the canola cultivars under evaluation are summarized in Table 2.

**Table 2:** Properties of canola genotypes used in the experiment.

Canola genotypes	Origin	Type of pollination	Growth type	Oil quality
Agamax	Germany	Hybrid	Spring	00
Hyola4815	Australia	Hybrid	Spring	00
Hyola50	Australia	Hybrid	Spring	00
Hyola401	Australia	Hybrid	Spring	00
Safi6	Iran	Open pollination	Spring	00
Zabol9	Iran	Open pollination	Spring	00
Zabol13	Iran	Open pollination	Spring	00

### 2.2 Experimental design

The study was implemented as strip plots within a randomized complete block design with three replications over two cropping seasons (2017–2018 and 2018–2019). Seven domestic and international canola cultivars (Agamax, Hyola 4815, Hyola 50, Hyola 401, Safi 6, Zabol 9, and Zabol 13) were evaluated across six sowing dates (September 23, October 7, October 22, November 6, November 21, and December 6).

### 2.3 Experiment details

Prior to sowing, 100 kg ha^−1^ of potassium from potassium sulfate and 70 kg ha^−1^ of phosphorus from triple superphosphate were incorporated into the soil. Nitrogen fertilizer amounting to 300 kg ha^−1^ from urea was applied in three equal portions: one-third before sowing, one-third at stem elongation (BBCH scale: 30), and one-third at flowering (BBCH scale: 61). For weed control, trifluralin herbicide was applied at 2 l ha^−1^ as a pre-sowing soil incorporation, followed by the creation of planting rows 75 cm apart using a furrower. Based on prior research, the planting arrangement consisted of two rows on a 75 cm ridge with a plant density of 60 plants per square meter (Kalantar Ahmadi 2014a; Kalantar Ahmadi 2014b). Crop management practices and weed control were carried out as required during the growing season. Each experimental plot covered an area of 12 m^2^. Irrigation was performed using furrows based on 70 mm evaporation from a Class A pan, with 600 cubic meters of water per hectare applied during each irrigation event. Mean and maximum air temperature during silique formation and seed filling period of canola genotypes in different sowing dates shown in Table 3.

**Table 3:** Mean and maximum air temperature during silique formation and seed filling period of canola genotypes in sowing date treatments (2017–2018 and 2018–2019).

Canola genotypes															
		Agamax		Hyola4815		Hyola50		Hyola401		Safi6		Zabol9		Zabol13	
Sowing dates	Temperature (°c)	2017–18	2018–19	2017–18	2018–19	2017–18	2018–19	2017–18	2018–19	2017–18	2018–19	2017–18	2018–19	2017–18	2018–19
Sep. 23	Max	25.13	21.91	21.34	19.18	25.38	22.47	24.68	21.73	25.08	22.24	25.08	22.33	25.08	22.33
	Mean	17.98	15.83	14.78	13.28	18.27	16.33	17.55	15.66	17.93	16.18	17.93	16.25	17.93	16.25
Oct. 7	Max	24.02	22.17	19.93	19.17	28.31	22.76	26.32	22.19	27.16	22.66	27.16	22.84	27.16	22.84
	Mean	16.84	16.18	13	13.31	21	16.49	18.94	16.13	19.84	16.44	19.87	16.57	19.87	16.57
Oct. 22	Max	25.62	22.16	21.37	19.66	27.37	23.44	26.75	22.5	26.79	23.04	26.75	23.04	26.75	23.04
	Mean	18.34	16.44	14.79	13.62	20.08	16.98	19.42	16.35	19.42	16.68	19.44	16.68	19.44	16.68
Nov. 6	Max	30.29	23.44	20.68	19.54	31.06	24.07	27.9	22.99	30.16	23.6	30.55	23.6	30.55	23.6
	Mean	22.33	16.98	14.34	13.63	23.22	17.52	21.68	16.65	22.18	10.62	22.57	10.62	22.57	10.62
Nov. 21	Max	30.78	26.7	22.99	20.47	31.05	27.89	30.99	26.68	31.12	27.03	31.21	27.03	31.21	27.03
	Mean	22.76	19.92	16.33	14.45	23.06	21.01	23.09	20	23.16	20.23	23.25	20.23	23.25	20.23
Dec. 6	Max	31.15	27.84	24.53	20.68	31.82	29.4	31.61	27.36	31.98	27.75	31.86	27.75	31.86	27.75
	Mean	23.17	20.95	18.01	14.65	24.16	22.05	23.66	20.55	24.09	20.94	24.07	20.94	24.07	20.94

### 2.4 Agronomic traits

During the physiological maturity stage (BBCH scale: 89), four central rows from each plot were harvested separately over an area of 6 m^2^ to assess seed yield. Ten plants were randomly selected to evaluate the number of siliques per plant.

### 2.5 Oil content and fatty acid composition

To assess the oil content in the seeds, a 5-g sample from each treatment group was chosen and analyzed using the NMR technique at the Seed Improvement Research Institute laboratory in Karaj. The composition of fatty acids was evaluated through gas chromatography (GS: Agilent Technologies, Santa Clara, CA, US), following the methodology established by Azadmard-Damirchi et al. [22]. Fatty acid methyl esters were produced from the oil samples. For sample preparation, 2 ml of 0.01 M NaOH in methanol was combined with an oil sample dissolved in 0.5 ml of hexane, and this mixture was incubated in a water bath at 60 °C for 10 min. Afterward, 20 % BF3 in methanol was introduced, and the sample remained in the water bath at 60 °C for another 10 min. Once cooled under running water, 2 ml of sodium chloride (20 % w/v) and 1 ml of hexane were added to the mixture. This blend was thoroughly mixed, and then centrifugation was used to separate the hexane layer containing the fatty acid methyl esters. The analysis of these esters was performed via GC as detailed by Azadmard-Damirchi and Dutta [23]. Glucosinolates were measured using a spectrophotometer equipped with a CP-Sil 88 capillary column that is 50-m long, has an internal diameter of 0.25 mm, and features a static thickness of 0.2 μm [24].

### 2.6 Statistical analysis

After conducting Bartlett's test in order to supply the homogeneity of the test variance in each year, the combined analysis of variance (ANOVA) was conducted using SAS software (version 9.2). The least significant difference (LSD) was used to determine the comparison of means at p < 0.05. Excell software was used to draw the graphs. The regression relationship (Stepwise method) between seed yield with phenological characteristics (Flowering initiation, flowering duration period, growth period duration) and temperature (Max, mean and min) was calculated by SPSS software. Cluster analysis was done by method of UPGMA (Euclidean) using Past software (Version 4.03).

## 3 Results

Based on combined analysis of variance results, the simple effect of year (except stearic acid), sowing date and genotype were significant on all studied traits. The interaction of year × sowing date was significant on start flowering, growth duration period, seed yield, palmitic acid, stearic acid, linoleic acid, linolenic acid and erucic acid. The interaction of sowing date × genotype, except for oil content, has a significant effect on other studied traits. Amount of growth duration period, seed yield, stearic acid, linolenic acid and erucic acid were impacted by the three-way interaction of year × sowing date × genotype (Table 4).

**Table 4:** Combined analysis of variance for studied traits as affected by sowing date (S) and genotype (G) during two years (2017–2018 and 2018–2019) in Dezful, Iran.

S.O.V	df	Flowering initiation	Flowering duration period	Growth period duration	Seed yield	Oil content	Palmitic acid	Stearic acid	Oleic acid	Linoleic acid	Linolenic acid	Erucic acid	Glucosinolate content
Y	1	∗∗	∗∗	∗∗	∗∗	∗∗	∗∗	ns	∗∗	∗∗	∗∗	∗∗	∗∗
Y (block)	4	–	–	–	–	–	–	–	–	–	–	–	–
S	5	∗∗	∗∗	∗∗	∗∗	∗∗	∗∗	∗∗	∗∗	∗∗	∗∗	∗∗	∗∗
Y × S	5	∗∗	∗∗	∗∗	∗∗	ns	∗∗	∗∗	ns	∗	∗∗	∗∗	ns
Ea	20	–	–	–	–	–	–	–	–	–	–	∗	–
G	6	∗∗	∗∗	∗∗	∗∗	∗∗	∗∗	∗∗	∗∗	∗∗	∗∗	∗∗	∗∗
Y × G	6	ns	∗∗	∗∗	∗∗	ns	∗∗	∗∗	∗∗	ns	∗∗	∗∗	ns
Eb	24	–	–	–	–	–	–	–	–	–	–	–	–
S × G	30	∗∗	∗∗	∗∗	∗∗	ns	∗∗	∗∗	∗∗	∗∗	∗∗	∗∗	∗∗
Y × S × G	30	ns	ns	∗∗	∗∗	ns	ns	∗∗	ns	ns	∗∗	∗∗	ns
Ec	120	–	–	–	–	–	–	–	–	–	–	–	–
CV %		0.56	1.02	0.11	10.84	3.23	5.81	3.91	1.96	3.98	10.07	16.69	8.09

*ns* not significant, *∗* and*∗∗* significant at 5 % and 1 % probability level, respectively. Y: year, E: error, S: sowing date, G: genotype, CV coefficient of variation, df: degree of freedom.

### 3.1 Flowering initiation

The studied genotypes had various initiate flowering on different sowing dates. Number of days for genotypes to initiate flowering was affected by sowing date. The longest time between sowing and initiation of flowering (86 days after sowing) was observed on the fourth sowing date (Nov. 6) and Zabol9 and Hyola50 genotypes in the first year (Table 5). In the second year of the experiment, the longest time interval between sowing and initiation of flowering was also allocated to the fourth sowing date and Zabol9 (91 days after sowing) and Hyola50 (90 days after sowing) genotypes, but this time interval increased compared to the first year (Table 5). The shortest time between sowing and initiation of flowering was observed on the second sowing date (Oct. 7) and Hyola4815 genotype in the first (45 days after sowing) and second (39 days after sowing) year (Table 5).

**Table 5:** Mean comparisons of the two-way interaction of sowing date × genotype on flowering initiation, flowering duration period, growth duration, seed yield, and palmitic acid in each year of experiment (2017–2018 and 2018–2019).

		Flowering initiation (days after sowing)		Flowering duration period (days after sowing)		Growth period duration (day)		Seed yield (kg ha^−1^)		Palmitic acid (%)	
Sowing date	Genotype	2017–18	2018–19	2017–18	2018–19	2017–18	2018–19	2017–18	2018–19	2017–18	2018–19
Sep. 23	Agamax	68 kl	60t	47p	75m	207b	222a	3020.4a-g	1919.4d-g	5.04a-h	6.44e
	Hyola4815	58s	50z	50no	78l	153u	166w	2163.4i-p	1677.6f-j	5.29a-f	7.25bc
	Hyola50	69k	61s	58h	86e	208a	222a	3143a-d	2391.7b	4.71a-i	6.24ef
	Hyola401	61r	53y	52l-n	80j	202e	217e	2325.6f-o	1625g-j	5.56a-d	7.14c
	Safi6	66n	57w	63f	92b	204d	219c	3035.7a-f	1650g-j	5.93a	7.65 ab
	Zabol9	66mn	58v	75a	103a	204d	219c	3144a-d	1144.4lm	5.6a-c	6.5e
	Zabol13	67lm	58v	75a	103a	205c	220b	2946.7a-i	1625g-j	5.29a-f	6.59de
Oct. 7	Agamax	71ij	64q	36t	58s	204d	217e	3286.8 ab	1766.7e-h	4.81a-i	6.25ef
	Hyola4815	45w	39z	52lm	73n	150v	166w	1992.3l-q	1838.9d-g	5.13a-g	7.03c
	Hyola50	54t	47z	59g	81i	205c	218d	3141.3a-d	2888.9a	4.89a-i	6.21ef
	Hyola401	48v	42z	58h	79k	199h	212g	2289.5h-o	1669.4f-j	5.21a-g	8.05a
	Safi6	50u	44z	62f	84g	200g	216f	2896.9a-h	1655.6g-j	5.52a-e	7.61
	Zabol9	68 kl	62r	68d	89c	200g	216f	2829.8a-j	2344.4b	4.81a-i	6.27ef
	Zabol13	71ij	64q	66e	88d	200g	218d	2863.4a-i	2105.6b-d	4.34b-i	5.64gh
Oct. 22	Agamax	68 kl	65p	41rs	55u	201f	212g	3357a	1986.1c-e	4.71a-i	5.3hi
	Hyola4815	57s	54x	50o	64r	147w	159x	2317.9g-n	1625g-j	4.92a-i	6.31ef
	Hyola50	65no	62r	57hi	71p	201f	210h	3064.3a-e	1963.8c-f	4.54b-i	5.1i
	Hyola401	63pq	60t	57hi	71p	197k	206k	2478.8d-m	1552.8h-k	5.34a-f	6.92cd
	Safi6	61r	58v	51no	65q	197.3j	208j	2719.1a-k	1427.8i-l	5.64 ab	6.4e
	Zabol9	62qr	59u	69cd	83h	198i	208j	2499.6c-m	1702.8e-i	5.21a-g	5.76g
	Zabol13	64pq	61s	71b	85f	198i	209i	2365e-n	1669.4f-i	4.74a-i	4.92ij
Nov. 6	Agamax	84b	89c	44q	46x	199h	204l	3211.6a-c	1905.6d-g	4.18e-i	4.93ij
	Hyola4815	77f	82h	48p	50v	143x	148y	2273.2h-o	1288.9k-m	4.68a-i	5.94 fg
	Hyola50	86a	90b	55ij	58s	198i	203m	3244.6 ab	2233.3bc	4.19d-i	4.92ij
	Hyola401	82cd	87e	54jk	56t	190n	195p	2582.5b-l	1093.9mn	4.72a-i	5.94 fg
	Safi6	84b	89c	48p	50v	191m	196o	2717a-k	1955.6c-f	5.11a-g	5.69gh
	Zabol9	86a	91a	68d	70o	190n	195p	2579.3b-l	1041.7mn	4.73a-i	5.58gh
	Zabol13	83bc	88d	70bc	72o	192l	197n	2147.9j-p	1393.1k-l	4.18e-i	4.65jk
LSD		1.11	0.64	1.5	0.77	0.25	0.77	713.93	295.13	1.37	0.4

		Flowering initiation (days after sowing)		Flowering duration period (days after sowing)		Growth period duration (day)		Seed yield (kg ha^−1^)		Palmitic acid (%)	
Sowing date	Genotype	2017–18	2018–19	2017–18	2018–19	2017–18	2018–19	2017–18	2018–19	2017–18	2018–19
Nov. 21	Agamax	81de	85f	35t	38z	190n	195p	2315g-o	1261.1k-m	3.98f-i	4.05lm
	Hyola4815	70j	74m	40s	43z	135y	140z	2074.4k-q	815.4n-p	4.16e-i	5.01ij
	Hyola50	85a	89c	45q	48w	188o	193q	2515.3c-l	1172.2lm	4.02f-i	4.37 kl
	Hyola401	81de	85f	42r	45x	181r	186s	1486.5p-r	1183.3lm	4.77a-i	5.05ij
	Safi6	84b	88d	36t	39z	182q	187r	1762.4n-r	694.4pq	4.56a-i	4.06lm
	Zabol9	81de	85f	51m-o	54u	181r	186s	2433.8d-n	538.9p-r	4.82a-i	4.47k
	Zabol13	80e	84g	53 kl	56t	181r	186s	2237h-o	819.4n-p	4.28b-i	3.79m-o
Dec. 6	Agamax	73h	75l	23w	26z	184p	186s	1100r	1016.8m-o	3.73hi	3.49no
	Hyola4815	57s	59u	22wx	31z	127z	130z	1801.1m-r	559.2p-r	4.4b-i	5.02ij
	Hyola50	75g	77k	31v	39z	181r	183t	1700o-r	732.8o-q	3.9g-i	3.84mn
	Hyola401	76 fg	78j	24w	31z	173t	175v	1235.9r	695.2pq	4.1f-i	5.03ij
	Safi6	77f	79i	19y	26z	174s	176u	1400qr	455qr	4.24c-i	3.76m-o
	Zabol9	70j	72o	31v	38z	173t	175v	1755.6n-r	385.5r	4f-i	4.02lm
	Zabol13	72i	73n	33u	41z	174s	176u	1704.4o-r	587.2p-r	3.6i	3.42o
LSD		1.11	0.64	1.5	0.77	0.25	0.77	713.93	295.13	1.37	0.4

Means in each column, followed by similar letter(s) are not significantly different at 5 % probability level, using LSD test.

### 3.2 Flowering duration period

Mean comparison between treatments showed that the longest flowering duration period appointed to the first sowing date (Sep. 23) and Zabol9 as well as Zabol13 genotypes in the first (75 days) and second year (103 days). The minimum flowering duration period observed on the last sowing date and Sai6 genotype in the first (19 days) and second year (26 days) of the experiment (Table 5).

### 3.3 Growth duration period

In the first year, mean comparison of interaction sowing date × genotype revealed that the maximum growth duration (208 days) observed the first sowing date (Sep. 23) and Hyola50 genotype. The minimum ones (127 days) appointed to on the last sowing date (Dec. 6) and Hyola4815 genotype (Table 5). In the second year, Agamax genotype with 222 days on the first sowing date (Sep. 23) and Hyola4815 with 130 days on the last sowing date (Dec. 6) indicated the highest and the lowest of growth duration, respectively (Table 5).

### 3.4 Seed yield

In the first year, Agamax genotype achieved the highest seed yield (3357 kg ha^−1^) on Oct 7th, but in the following year the peak seed yield (2888.9 kg ha^−1^) belonged to the second sowing date (Oct. 7) and Hyola50 genotype (Table 5). The lowest seed yield were noted for Agamax (1100 kg ha-1) and Zabol9 (385 kg ha^−1^) genotypes when they were planted on Dec. 7 in the first and second year, respectively (Table 5). The analysis of regression between seed yield and temperature along with phenological traits indicated that growth period duration and flowering initiation had positive effect, but maximum temperature had negative effect on seed yield in the first year.

$${Seed\;yield}_{year\;1}=541.99+22.42\;Growth\;period\;duration-123.43\;\mathrm{Max}\;temperature+15.93\;Flowering\;initiation$$

There was a positive relationship between growth period duration and seed yield, as well as a negative relationship between seed yield and maximum temperature observed in the second year.

$${Seed\;yield}_{year\;2}=141.94+13.98\;Growth\;period\;duration-58.81\;\mathrm{Max}\;temperature$$

### 3.5 Oil content

**Fig. 2:**
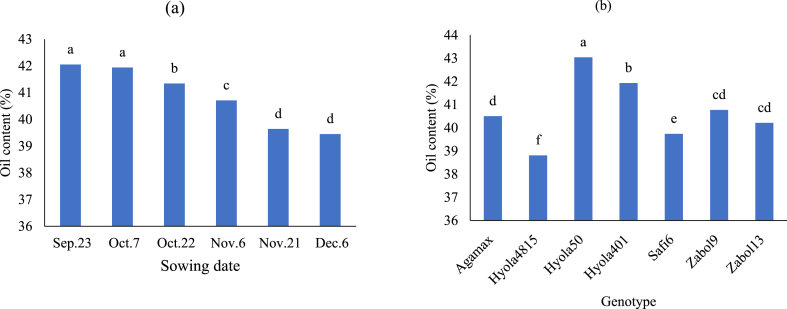
Mean comparison of sowing date (a) and genotype (b) for oil content of canola during two study years (2017–2018 and 2018–2019).

The average oil content varied significantly over the two years; specifically, it was higher in the first year at 41.11 % compared to 40.6 % in the second year (data not presented). The delay in sowing date resulted in a reduction of oil content. The results revealed that the maximum (42.05 %) and the minimum (39.45 %) oil content belonged to the first (Sep. 23) and the last (Dec. 6) sowing date during two years of the experiment (Fig. 2a). Between studied genotypes, Hyola50 and Hyola4815 produced the highest (43.04 %) and the lowest (38.81 %) oil content, respectively (Fig. 2b).

### 3.6 Palmitic acid

Delaying sowing dates led to a decrease in oil content. A comparison of means for the interaction between sowing date and genotype revealed that Safi6 produced the highest palmitic acid content (5.93 %) on the first sowing date (Sep. 23), while Zabol13 yielded the lowest amount (3.6 %) on the final sowing date (Dec. 6) during the first experimental year (Table 5). The results showed that the maximum palmitic acid (8.05 %) belonged to the second sowing date (Oct. 7) and Hyola401 genotype, while Zabol13 genotype produce the minimum palmitic acid (3.42 %) on the last sowing date in the second year (Table 5).

### 3.7 Stearic acid

The means comparison of two-way interaction of sowing date and genotype illustrated that the maximum amount of stearic acid (6.48 %) was appointed to the sowing date on Nov. 21 and Hyola401 genotype in the first year. In contrast, during the second year, Hyola401 again showed high levels of stearic acid at 5.61 % on Oct. 7 (Table 6). The lowest stearic acid (3.12 %) was found in Agamax and Zabol13 genotypes in the first sowing date (Sep. 23) in the first year and in the second year the minimum ones appointed to Zabol13 in sowing date Nov. 21 (Table 6).

**Table 6:** Mean comparisons of the two-way interaction of sowing date × genotype on stearic acid, oleic acid, linoleic acid, linolenic acid, erucic acid, and glucosinolate content in each year of experiment (2017–2018 and 2018–2019).

		Stearic acid (%)		Oleic acid (%)		Linoleic aicd (%)		Linolenic acid (%)		Erucic acid (%)		Glucosinolate content (μmol g^−1^)	
Sowing date	Genotype	2017–18	2018–19	2017–18	2018–19	2017–18	2018–19	2017–18	2018–19	2017–18	2018–19	2017–18	2018–19
Sep. 23	Agamax	3.12u	4.21a-d	54.2m-q	50.45no	23.25d-f	21.15ef	4.58e-h	3.36 ab	0.374bc	0.317a	3.32tu	2.7t
	Hyola4815	4.09n	5.05a-c	56.14h-l	52.67h-k	25.22bc	24.14bc	5.33bc	6.14a-c	0.202f-h	0.142d-i	4.93m-p	4.18l-o
	Hyola50	3.34st	4.18a-d	57.21f-i	51.19k-o	27.26a	26.17a	4.37f-j	5.61b-d	0.285d	0.231bc	3.33tu	2.91t
	Hyola401	4.23m	5.07a-c	52.92o-s	50.37no	26.14 ab	24.17bc	5.21cd	6.21 ab	0.388a-c	0.335a	4.04q-t	3.54p-s
	Safi6	3.36st	4.94a-c	52.66p-t	50.54no	22.95e-h	21.32ef	5.81 ab	6.81a	0.397a-c	0.354a	4.27o-s	3.64o-r
	Zabol9	3.33t	4.17a-d	49.6u	50.41no	23.16e-g	21.34ef	4.31f-j	6.35 ab	0.378a-c	0.335a	3.86r-t	3.49q-s
	Zabol13	3.12u	4.06b-d	50.8tu	50.02o	22.85e-h	21.06e-g	4.32f-j	6.23 ab	0.412 ab	0.360a	2.61u	3.06st
Oct. 7	Agamax	3.42s	4.23a-d	52.52q-t	51.36k-o	25.16bc	24.35 ab	4.32f-j	5.2de	0.295d	0.237bc	3.64st	3.22r-t
	Hyola4815	4.27m	5.04a-c	54.58k-o	51.3k-o	24.13c-e	23.15vc	5.13c-e	5.36c-e	0.152jk	0.083h-l	5.3l-n	4.48j-l
	Hyola50	3.52r	4.13a-d	56.14h-l	51.66j-o	26.91a	25.51 ab	4.03h-l	5.11d-f	0.237ef	0.183c-f	3.62nd	3.01st
	Hyola401	4.6j	5.61a	51.41r-u	50.81m-o	25.85 ab	24.36bc	5.92a	5.91b-d	0.365c	0.305 ab	4.52n-r	4.07l-p
	Safi6	3.64q	4.83a-d	50.88tu	50.81m-o	23.49d-f	21.36ef	5.3bc	6.17 ab	0.418a	0.363a	4.79n-q	4.28k-n
	Zabol9	3.77p	4.31a-d	52.06r-t	50.91l-o	22.54f-i	20.38f-h	4.16g-k	5.62b-d	0.379a-c	0.331a	4.23p-s	3.78n-q
	Zabol13	3.29t	3.94cd	51.99r-t	50.8m-o	22.24f-i	20.16f-i	3.85j-n	5.21de	0.406 ab	0.346a	4.34o-s	3.88m-q
Oct. 22	Agamax	3.6q	4.10b-d	53.16o-r	52.19j-m	24.22c-e	23.24cd	3.87j-n	4.62e-g	0.222e-g	0.117e-j	4.24p-s	3.86m-q
	Hyola4815	4.5k	5.05a-c	53.31n-r	50.65m-o	24.16c-e	22.18de	4.86c-f	4.72e-g	0.095lm	0.106g-l	6.3h-j	5.97hi
	Hyola50	3.9o	4.14a-d	58.44d-f	53.1g-j	24.73b-d	24.09bc	3.64k-o	4.66e-g	0.181h-j	0.072i-l	4.31o-s	3.81n-q
	Hyola401	4.92h	5.43 ab	54.13m-q	52.21j-m	24.22c-e	23.22cd	5.32bc	4.37f-h	0.280d	0.174c-g	5.12m-o	4.75jk
	Safi6	4.3m	4.85a-d	51.17s-u	52.74g-k	22.29f-i	21.36ef	4.65d-g	5.68b-d	0.296d	0.185c-e	5.67j-m	4.92j
	Zabol9	4.23m	4.52a-d	53.19o-r	52.21j-m	21.21ij	20.38f-h	4.07h-k	5.15d-f	0.26de	0.154d-h	5.35k-n	4.6j-l
	Zabol13	3.5r	3.82cd	53.18o-r	52.53i-l	21.06ij	20.16f-i	3.41m-r	4.66e-g	0.281d	0.172c-g	4.85m-q	4.4j-m
LSD		0.08	1.48	1.91	1.71	1.55	1.66	0.57	0.8	0.04	0.07	0.86	0.54

		Stearic acid (%)		Oleic acid (%)		Linoleic aicd (%)		Linolenic acid (%)		Erucic acid (%)		Glucosinolate content (μmol g^−1^)	
Sowing date	Genotype	2017–18	2018–19	2017–18	2018–19	2017–18	2018–19	2017–18	2018–19	2017–18	2018–19	2017–18	2018–19
Nov. 6	Agamax	4.31m	4.12a-d	55.19j-n	53.3g-j	22.17f-i	21.37ef	3.17o-r	4.13gh	0.159i-k	0.073i-l	6.1i-l	5.88hi
	Hyola4815	5.3f	5.07a-c	54.46k-p	52.03j-n	21.63g-j	21.67ef	4.15g-k	4.25gh	0.040o-q	0.199cd	7.49b-e	7.03de
	Hyola50	4.08n	4.16a-d	57.49e-h	55.11ef	23.18d-g	23.18cd	3.49l-p	4.27gh	0.097lm	0.057j-l	5.35k-n	4.89j
	Hyola401	5.64e	4.45 ab	56.36g-k	54.29f-h	21.54h-j	20.38f-h	4.52f-i	4.22gh	0.128 kl	0.085h-l	6.21i-k	5.59i
	Safi6	4.77i	4.67a-d	55.56i-m	54.22f-i	20.2jk	19.56g-j	3.92j-n	5.15d-f	0.228e-g	0.138d-i	7.2c-f	6.74e-g
	Zabol9	4.42l	4.49a-d	55.2j-n	54f-i	19.15 kl	18.37jk	3.85j-n	4.7e-g	0.194g-i	0.102g-l	6.32g-j	6.03hi
	Zabol13	3.84op	3.84cd	54.35l-q	53.97f-i	18.29lm	17.62k-m	3.13o-r	4.35f-h	0.199f-h	0.111f-k	5.72j-m	5.54i
Nov. 21	Agamax	4.89h	3.88cd	57.34f-i	55.14ef	19.31 kl	18.37jk	3.07o-r	3.62h-k	0.088l-n	0.078i-l	6.82e-i	6.26gh
	Hyola4815	5.68e	4.72a-d	59.6f-j	54.43 fg	18.12lm	18.04j-l	3.98i-m	4.11gh	0.028pq	0.063j-l	8.34 ab	7.54b-d
	Hyola50	4.6j	3.8cd	61.21bc	58.23bc	20.3jk	19.35h-j	3.23o-r	3.15i-l	0.052n-q	0.059j-l	6.48f-j	6.35f-h
	Hyola401	6.48a	5.28a-c	59.38c-e	56.53c-e	19.43 kl	18.62i-k	4.36f-j	4.11gh	0.065m-p	0.072i-l	7.12d-h	6.91e
	Safi6	5.88c	4.5a-d	58.38ef	56.38de	18.38lm	18.12j-l	3.36n-r	3.18i-l	0.083mn	0.08i-l	7.94a-d	7.72b
	Zabol9	4.61j	3.9cd	58.32ef	56.49de	17.24m-o	17.16k-n	3.43m-q	2.85k-m	0.063m-p	0.054j-l	7.18d-g	6.87ef
	Zabol13	4.11n	3.41d	58.23e-g	56.75b-e	17.23m-o	16.63l-o	3.04p-r	2.94j-m	0.069m-o	0.072i-l	6.83e-i	6.19h
Dec. 6	Agamax	5.17g	4.01b-d	60.31b-d	57.4b-d	16.31op	15.78n-p	2.87qr	2.6lm	0.022q	0.036l	8.06a-c	7.69bc
	Hyola4815	5.89c	4.85a-d	58.3ef	55.48ef	17.92l-n	16.3m-o	3.85j-n	3.67h-j	0.016q	0.044j-l	8.41a	7.99 ab
	Hyola50	4.9h	3.98c-d	63.38a	60.89a	17.46m-o	16.29m-o	3.1o-r	3.06j-l	0.015q	0.052j-l	7.14d-h	6.85ef
	Hyola401	5.79d	4.87a-d	61.33b	58.36b	16.46n-p	15.08op	3.93j-n	3.93g-i	0.027pq	0.039 kl	7.75a-d	7.68bc
	Safi6	6.39b	4.74a-d	59.41c-e	57.63b-d	16.16op	15.82n-p	3.12o-r	2.56lm	0.027pq	0.041 kl	8.44a	8.35a
	Zabol9	4.89h	3.97b-d	58.41d-f	57.58b-d	16.27op	15.32op	3.04p-r	2.25m	0.020q	0.037 kl	8.31 ab	8.04 ab
	Zabol13	4.87h	3.86cd	59.36c-e	57.73b-d	15.6p	14.62p	2.84r	2.15m	0.021q	0.037 kl	7.88a-d	7.15c-e
LSD		0.08	1.48	1.91	1.71	1.55	1.66	0.57	0.8	0.04	0.07	0.86	0.54

Means in each column, followed by similar letter(s) are not significantly different at 5 % probability level, using LSD test.

### 3.8 Oleic acid

Based on mean comparison results, the canola genotypes responded differently to sowing date regarding oleic acid, so that that the highest amount of oleic acid observed in Hyola50 in the last sowing date (Dec. 6) in the first (63.38 %) and second year (60.89 %). Zabol9 genotype had the lowest oleic acid (49.6 %) on the first sowing date (Sep. 23) in the first year, while in the second year the minimum ones (50.02 %) belonged to the first sowing date (Sep. 23) and Zabol13 genotype (Table 6).

### 3.9 Linoleic acid

A notable difference was found in how the studied genotypes responded to changes in sowing dates regarding linoleic acid content. The average comparison indicated that in both the first (27.26 %) and second (26.17 %) years, the Hyola50 genotype produced the highest levels of linoleic acid on the initial sowing date (Table 6). Conversely, on the last sowing date (Dec. 6), the lowest linoleic acid levels were recorded for the Zabol13 genotype in both the first (15.6 %) and second years (14.62 %) (Table 6).

### 3.10 Linolenic acid

The maximum amount of linolenic acid (5.92 %) belonged Hyola401 in the second sowing date (Oct. 7) in the first year, while Safi6 produced the maximum (6.81 %) ones when planted on Sep. 23 in the second year (Table 6). The minimum values were obtained from Zabol13 genotype with averages of 2.15 and 2.25 % when planted on Dec. 6 in the first and second year, respectively (Table 6).

### 3.11 Erucic acid

When compared treatments in the first year, Safi6 genotype produced the highest erucic acid (0.418 %) on the second sowing date (Oct. 7), as well as that Hyola50 and Hyola4815 produced the lowsest erucic acid by 0.015 and 0.016 %, respectively on the last sowing date (Dec. 6) (Table 6). The average erucic acid of Safi6 on Oct. 7 was 0.363 %, while the amount of this trait for Agamax genotype grown on the last sowing date (Dec. 6) was the minimum amout (0.036 %) in the second year (Table 6).

### 3.12 Glucosinolate

During two years, canola genotypes significantly differed in the amount of glucosinolate in sowing dates. The studied genotypes had a significant difference in the amount of glucosinolate in sowing date during two years of the experiment. In the first year, based on two-way interaction of sowing date × genotype, the maximum amount of glucosinolate detected to Safi6 (8.44 μmol g^−1^) and Hyola4815 (8.41 μmol g^−1^) on the last sowing date (Dec. 6), while Zabol13 genotype produced the minimum amount (2.61 μmol g-1) of this trait on Sep. 23 (Table 6). In the second year, the highest (8.35 μmol g^−1^) glucosinolate content appointed to the last sowing date (Dec. 6) and Safi6 genotype, while Agamax genotype with an average of 2.7 μmol g^−1^ showed the lowest ones (Table 6).

## 4 Discussion

The variations observed in the studied traits can be linked to differences in climatic conditions (Temperature, humidity, rainfall, etc), and the interactions among these factors throughout the growth period (Fig. 1a and b), particularly temperature during flowering and seed filling. This study examined temperature and sowing date as key environmental factors influencing plant adaptation to their surroundings (Table 3). Notably, differences in temperature due to changes in sowing date and the length of phenological stages are significant. The timing of flowering initiation and the duration of flowering varied among genotypes in different sowing dates during both years. In the first and second year of the experiment, Zabol9 and Hyola50 genotypes had the longest time between sowing and flowering initiation on the fourth sowing date (Table 5). The shortest interval between sowing and flowering initiation was observed for the Hyola4815 genotype on the second sowing date; this interval occurred six days earlier in the second year compared to the first year, highlighting the influence of environmental conditions on genotype responses. In terms of flowering duration, Zabol9 and Zabol13 genotypes exhibited the longest flowering periods during both years of the study. However, higher temperatures in the first year of the experiment compared to the second year led to a reduction in the flowering duration period (Fig. 1). These results were consistent with some researches about the effect of environment on rapeseed flowering [25,26]. The postponement of the sowing date resulted in a shorter flowering duration across all genotypes evaluated. This delay in planting coincided with rising environmental temperatures during the flowering phase, which contributed to a reduction in the length of the flowering period. Reduction the flowering duration period means that there is less opportunity for the formation of siliques, and this can lead to a decrease in seed yield [4,27]. Delayed sowing reduced the growth period duration in all genotypes tested. Hyola4815 genotype was the earliest genotype and had the shortest growth period in all sowing dates (Table 5). In general, delayed planting led to a reduction in the growth period, emphasizing the importance of selecting appropriate sowing dates for optimal plant development and seed yield in canola. Khayat et al. [9] stated that with the onset of flowering, the process of photosynthesis decreases significantly, and the plant becomes sensitive to environmental stresses such as high temperatures. Improper sowing dates leads to reduce the growth period duration in canola genotypes, and terminal heat stress reduce seed filling period and transfer of photosynthetic materials to seeds, ultimately reducing seed yield. Flowering is one of the important stage to transition to the reproductive phase, which is controlled by different genes, and under stress conditions, the plant uses different mechanisms to change flowering in order to maintain survival [28].

Environmental conditions, including high temperatures during vegetative and reproductive periods, are determinants of seed yield [4,29,30]. The response of the genotypes under investigation to climatic conditions differed between the two years of the experiment. Despite the fact that the siliquing and seed-filling periods of the genotype Hyola4815 did not coincide with high temperatures (Table 3), its seed yield was lower than that of the other genotypes. This can be attributed to the inherently low yield potential of this genotype. Climatic variations between the two years of the experiment led to changes in the yield of canola genotypes, with most genotypes exhibiting lower seed yield in the second year. The reduction in seed yield of the Hyola50 genotype was less compared to other varieties (Table 5), suggesting that this genotype has greater yield stability under changing weather conditions than the other genotypes. Although temperatures during the siliquing and seed-filling periods were lower in the second year compared to the first year (Table 3), and higher seed yields were expected, heavy rainfall (104 mm over two consecutive days) in late-March of the second year (Fig. 1), along with strong winds, caused lodging of plants. This led to silique shattering due to temperature fluctuations, seed shedding, and ultimately reduced seed yield. Furthermore, plant lodging stimulated a re-flowering process, which also contributed to the reduction in seed yield. The lodged plants not only failed to return to their original upright position but also initiated new flowering. The newly formed flowers did not positively affect yield; instead, they were damaged by high temperatures and failed to produce seeds. Consequently, assimilates that should have been allocated to seed filling were diverted to support the newly formed flowers, leading to further reductions in seed yield. The negative impact of lodging on seed yield through disruption of the accumulation and distribution of photosynthates and nutrients has been reported [31,32]. Moreover, the timing of lodging occurrence affects yield loss, with lodging after flowering causing more severe yield reductions [[33], [34], [35]]. The detrimental effects of lodging on grain yield are more pronounced during post-flowering stages.

**Fig. 3:**
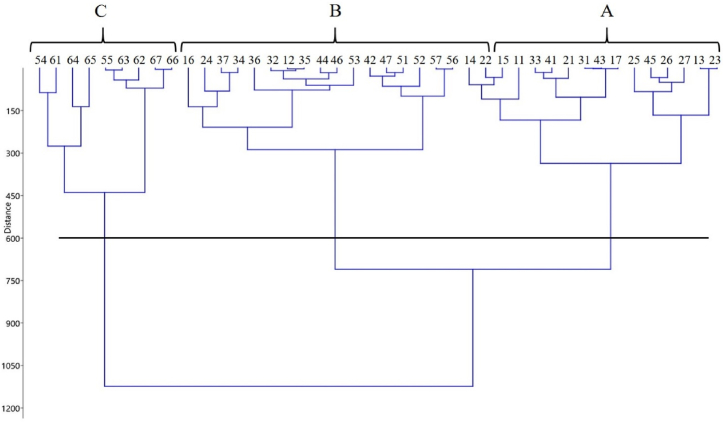
The cluster analysis of canola genotypes in different sowing date by method of UPGMA (Euclidean) in 2017–2018. 11: S1G1, 12: S1G2, 13: S1G3, 14: S1G4, 15: S1G5, 16: S1G6, 17: S1G7, 21: S2G1, 22: S2G2, 23: S2G3, 24: S2G4, 25: S2G5, 26: S2G6, 27: S2G7, 31: S3G1, 32: S3G2, 33: S3G3, 34: S3G4, 35: S3G5, 36: S3G6, 37: S3G7, 41: S4G1, 42: S4G2, 43: S4G3, 44: S4G4, 45: S4G5, 46: S4G6, 47: S4G7, 51: S5G1, 52: S5G2, 53: S5G3, 54: S5G4, 55: S5G5, 56: S5G6, 57: S5G7, 61: S6G1, 62: S6G2, 63: S6G3, 64: S6G4, 65: S6G5, 66: S6G6, 67: S6G7. Note: S1, S2 and S3 indicate different sowing dates (Sep. 23, Oct. 7, Oct. 22, Nov. 6, N. 21 and Dec. 6, respectively) while G1, G2, G3, G4, G5, G6 and G7 show Agamax, Hyola4815, Hyola50, Hyola401, Safi6, Zabol9, and Zabol13 genotypes, respectively.

The results of cluster analysis using the UPGMA method for seed yield across different sowing dates for seven genotypes studied in the first year indicated that the results could be grouped into three main clusters (Fig. 3). Group A included the first, second, and occasionally third sowing dates. In this group, the genotype Agamax had the highest grain yield (3357.04 kg ha^−1^) on the third planting date (Oct. 22), followed by Agamax on the first sowing date (Sept. 23) and Hyola50 on the fourth sowing date, ranking second and third, respectively (Table 5). Group B generally included the third, fourth, and fifth sowing dates, indicating that intermediate sowing dates were represented in this group (Fig. 3). The results showed that genotype Hyola401 had the highest seed yield (2582.52 kg ha^−1^) on the fourth planting date (Nov. 6), followed by Zabol9 on the same date and Hyola50 on the Nov. 21 (Table 5). Group C included the sowing dates of Nov. 21 and Dec. 6 (Fig. 3), with genotype Hyola4815 achieving the highest seed yield (1801.11 kg ha^−1^) on Dec. 6. The mean seed yield in groups A, B, and C in the first year (Fig. 3) was 3038, 2328, and 1592 kg ha^−1^, respectively. The average temperatures on the sowing dates of Sep. 23, Oct. 7, and Oct. 22 were 25.53, 25.72, and 25.91 °C, respectively and from the sowing date of Nov. 6 onwards, the maximum temperature rose above 28 °C. Therefore, the reason that the first (Sep. 23) and second (Oct. 7) sowing dates were predominantly in group A can be attributed to the lower temperatures during these planting periods throughout the seed filling period. Kalantarahmadi & Daneshian [4] also stated that for each unit increase in temperature above 28 °C during seed filling period, seed yield decreases by 340 kg ha^−1^.

**Fig. 4:**
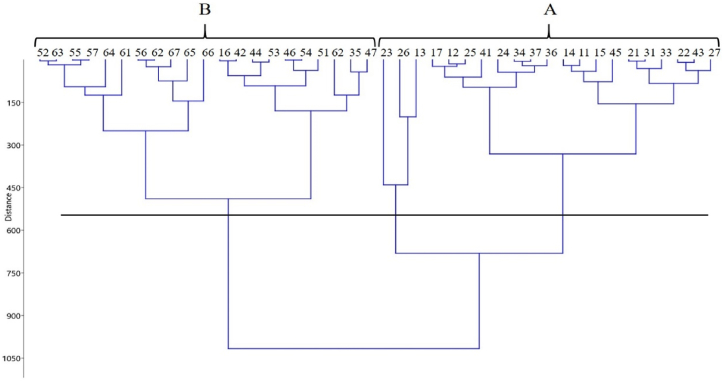
The cluster analysis of canola genotypes in different sowing date by method of UPGMA (Euclidean) in 2018–2019. 11: S1G1, 12: S1G2, 13: S1G3, 14: S1G4, 15: S1G5, 16: S1G6, 17: S1G7, 21: S2G1, 22: S2G2, 23: S2G3, 24: S2G4, 25: S2G5, 26: S2G6, 27: S2G7, 31: S3G1, 32: S3G2, 33: S3G3, 34: S3G4, 35: S3G5, 36: S3G6, 37: S3G7, 41: S4G1, 42: S4G2, 43: S4G3, 44: S4G4, 45: S4G5, 46: S4G6, 47: S4G7, 51: S5G1, 52: S5G2, 53: S5G3, 54: S5G4, 55: S5G5, 56: S5G6, 57: S5G7, 61: S6G1, 62: S6G2, 63: S6G3, 64: S6G4, 65: S6G5, 66: S6G6, 67: S6G7. Note: S1, S2 and S3 indicate different sowing dates (Sep. 23, Oct. 7, Oct. 22, Nov. 6, N. 21 and Dec. 6, respectively) while G1, G2, G3, G4, G5, G6 and G7 show Agamax, Hyola4815, Hyola50, Hyola401, Safi6, Zabol9, and Zabol13 genotypes, respectively.

Cluster analysis in the second year showed that the obtained results could be grouped into two main clusters (Fig. 4). Group A included the first, second, third and occasionally forth sowing dates. In this group, Hyola50 in the second sowing date (Oct. 7) had the maximum seed yield (2781.48 kg ha^−1^), followed by Hyola50 and Zabol9 on sowing dates Sep. 23 and Oct. 7, respectively (Table 5). Group B encompassed sowing dates of Nov. 6, Nov. 21 and Dec. 6. The mean seed yield in Groups A and B in the second year (Fig. 4) were recorded at 1903 and 849 kg ha^−1^, respectively. Despite experiencing heavy rainfall and lodging that reduced seed yields in the second year of the study, early sowing dates within Group A still produced higher yields. Based on the results from the two years of testing, it can be stated that considering the climatic conditions of the region and the tested genotypes, delayed sowing dates (after Nov. 6) are not recommended.

Comparison of average sowing dates in two years of study showed that delaying sowing date reducing the silique period, and seed filling period led to a decrease in oil content (Fig. 2a). Oil content is affected by temperature during the seed filling stage; increased temperatures during this time lead to a decrease in seed oil content. Environmental stresses such as elevated temperatures during seed filling hinder the production of precursors necessary for triacylglycerol synthesis [3] and fatty acid biosynthesis activities [36], ultimately resulting in lower seed oil content [14]. Heat stress also reduces oil content due to disruptions in oil biosynthesis [36]. Climatic conditions and sowing date significantly affect oil content, as plant productivity depends on aligning growth stages with environmental conditions. The difference in seed oil content in the evaluated genotypes can be attributed to their genetic differences and the effect of environmental factors on the amount of oil is less compared to other traits [37,38]. Photosynthesis by green seeds and siliques induces the formation of essential components of the fatty acid biosynthesis pathway [20] and control of this pathway is carried out by the transcription factor BnWRI1 [19]. Heat stress also reduces the amount of oil by inhibiting photosynthesis and reducing the expression of many genes [20].

Palmitic acid and stearic acid are recognized as two primary unsaturated fatty acids [39]. Climatic conditions impacted palmitic acid levels, which were higher in the second year (5.54 %) compared to the first year (4.7 %), while stearic acid levels remained consistent between years one (4.44 %) and two (4.46 %), showing no significant difference (data not shown). Although some research indicates an increase in saturated fatty acids under high-temperature conditions, our findings revealed that elevated temperatures during seed filling in the first year led to a decrease in palmitic acid compared to those observed in the second year (data not shown), with varying responses among genotypes. Genotype Hyola4815, being one of the earliest genotypes with a shorter growth period, had high palmitic acid content throughout all sowing dates compared to the late-maturing genotype Hyola50, indicating the significant role of genetic traits in determining palmitic acid amount. The higher amount of stearic acid in Hyola401 in the first and second year of the experiment indicates the genetic characteristics of this genotype regarding the high level of stearic acid, but considering the fact that it has changed in the two years of the experiment with different sowing dates, it indicates the effect of environmental factors on the amount of stearic acid.

While genetic traits influence oil quantity and fatty acid composition, temperature conditions during seed filling also play a crucial role [3,4]. Enzymes involved in oil biosynthesis are sensitive to high temperatures; thus, an increase in temperature can lead to reduced oil content [40]. Some studies have found no impact on unsaturated fatty acid levels due to temperature variations [3], while others report an increase in oleic acid alongside a decrease in linoleic acid [41]. The degree and timing of temperature effects on fatty acids depend on both intensity and timing of heat exposure; current results align with other studies indicating that delayed sowing dates can positively affect oleic acid levels while reducing linoleic acid during seed filling (Table 6) [4,41]. The rise in oleic acid at elevated temperatures may stem from limitations on converting oleic acid into linoleic acid as a precursor. This increase could be linked to restricted conversion processes under high-temperature conditions [42]. The enzyme oleate desaturase is responsible for converting oleic acid into linoleic acid through cytosolic desaturation; variations in its activity may explain shifts in the oleic/linoleic ratio under drought and heat stress conditions [43]. Linoleic acid is produced from oleic acid desaturation [44], with each step from oleic to linoleic being potentially influenced by both temperature duration and genetic factors [45]. Furthermore, different genotypes exhibit varying sensitivities within their fatty acids' biosynthesis metabolic pathways when exposed to temperature changes [45].

Irrigation can also mitigate heat stress and influence the activity of the enzyme oleate desaturase, thereby affecting fatty acid levels [46]. Elevated temperatures during the seed filling stage result in a rise in oleic acid levels while causing a decline in linolenic acid. Moreover, the duration of high temperatures has a greater impact on fatty acid composition compared to the timing of their occurrence [41]. Although the responses of the tested genotypes regarding changes in oleic and linolenic acids varied across different sowing dates, generally, in delayed sowings where temperatures were higher during the seed filling period, oleic acid increased while linolenic acid decreased. Research has also indicated that high temperatures positively influence the increase of oleic acid more significantly than that of linolenic acid [47]. Fluctuations in temperature during this period lead to alterations in fatty acid composition [48,49], with oleic and linolenic acids in canola being particularly responsive to these temperature variations [50]. The final amount of linolenic acid is determined by the desaturation rate of C18:1 to produce C18:2, followed by the conversion of C18:2 into C18:3 [47]. Variations in the fatty acid profiles among rapeseed cultivars can be attributed to their genetic diversity, and differences observed among the genotypes tested align with findings from other studies [[39], [51], [52]].

Erucic acid is a significant fatty acid concerning its effects on human health, and its concentration is affected by both genetic factors and environmental conditions [4,53]. Delay in sowing date was found to reduce erucic acid levels in the current experiment, with genotype responses differing, indicating environmental factors influence on erucic acid. These results align with Gharechaei et al. [46] findings on reduced erucic acid in late plantings.

An increase in glucosinolate content within seeds negatively impacts the quality and nutritional value of rapeseed meal [54], which is also influenced by genetic and environmental variables [55]. Unlike erucic acid, glucosinolate levels increase with delayed sowing dates, consistent with findings by other researches [4,53]. It seems that when the seeds are in the filling stage and the plant is faced with heat stress [4], the amount of glucosinolates in vegetative tissues and siliques then remobilized to seeds and finally its amount increases in the seed [56]. Halting mineral and metabolite transfer to seeds also stops glucosinolate transport [57]. Approximately 78 loci related to glucosinolates have been identified, with 36 genes involved in glucosinolates biosynthesis. The key gene is BnaA03g40190D, determining high leaf glucosinolates and low seed glucosinolates, contributing to increased resistance of rapeseed genotypes against pests and diseases [59]. In this study, the levels of erucic acid (below 2 %) and glucosinolate (under 30 μmol g−1) were found to be within acceptable limits [7].

## 5 Conclusion

Achieving optimal seed yield in canola necessitates the plant's adaptation to key environmental parameters, including temperature, humidity, and both the spatial and temporal distribution of rainfall. Hence, the timing of sowing, in conjunction with other environmental factors, represents a critical agronomic determinant influencing the plant's adaptation to its surroundings, ultimately shaping both the yield and quality of canola production. This study underscores the pivotal role of temperature in modulating seed yield and the composition of fatty acids. Postponing the sowing date (from Sep. 23 to Dec. 6) and elevating the mean temperature from 17.98 °C to 23.17 °C in the first year, and from 15.83 °C to 20.95 °C in the second year, during the silique formation and seed filling stages, induced alterations in both seed yield and fatty acid composition across the examined genotypes. The Hyola50 genotype exhibited a comparatively lower reduction in seed yield than the other assessed genotypes. The differences in the responses of the studied genotypes indicate genetic diversity in reaction to temperature and environmental conditions. Therefore, heat tolerance is a trait that should be considered in breeding programs due to climate change.

## CRediT authorship contribution statement

**Seyed Ahmad Kalantar Ahmadi:** Writing – original draft, Software, Project administration, Methodology, Investigation, Formal analysis, Conceptualization. **Mohsen Sarhangi:** Writing – review & editing, Software.

## Data availability statement

Data is not available for the statement.

## Additional information

No additional information is available for this paper.

## Funding statement

There was no source of funding for the manuscript from any public, private, or non-profit funding bodies was allocated to this study.

## Declaration of competing interest

The authors declare that they have no known competing financial interests or personal relationships that could have appeared to influence the work reported in this paper.
