# Supplementary material for: Optimizing sowing date for enhanced heat stress tolerance in canola (Brassica napus L.): Investigating impacts on seed yield, oil content, and fatty acids composition
Source: Heliyon. 2025 Jan 21;11(2):e42138. doi: 10.1016/j.heliyon.2025.e42138 (PMC11804544; doi:10.1016/j.heliyon.2025.e42138)
Supplement: Multimedia component 1 [file mmc1.pdf]

Similarity Report

iThenticate

15%

Optimizing Sowing Date for Enhanced Heat Stress...

As of: Jan 20, 2025 12:29:56 PM

5,946 words - 93 matches - 34 sources

VIEW

VIEW

paper text:

Abstract Environmental conditions, including temperature and the occurrence of phenological stages at the optimum temperature, are effective factors

2 on seed yield, oil content and fatty acids

. An

4 experiment was carried out as strip block based on randomized completed block design with three replications in Safiabad Agricultural and Natural Resources Research and Education Center of Dezful during two cropping seasons

(2017-2019). Vertical factors consisted of six levels of sowing date (23 Sep, Oct 7, 22 Oct, Nov 6, 21 Nov, Dec 6), and horizontal factors were seven genotypes (Agamx, Hyola4815, Hyola50, Hyola401, Safi6, Zabol9 and Zabol13). Elevated temperatures resulting from postponed sowing dates during the silique formation and seed filling phases led to a decline in

1 seed yield, oil content, and modifications in the fatty acid composition of

the studied canola genotypes. The highest (43.04%) and lowest (38.81%) oil content over the two years of testing were attributed to the genotypes Hyola50 and Hyola4815, respectively. Postponing the sowing date contributed to a higher accumulation of oleic acid in the examined genotypes. The maximum oleic acid content (62.14%) was observed on Dec. 6 for the genotype Hyola50, while the minimum oleic acid content (50%) was recorded on Sep. 23 for the genotype Zabol9 during two years of the experiment. Variations in climatic conditions across the two experimental years elicited distinct responses in the studied genotypes based on the sowing date. In the first year, Agamax genotype

1 produced the highest seed yield (3357 kg ha-1

) on Oct 7th, but in the second year

4 the highest seed yield (2888.9 kg ha-1) belonged to the second sowing date

(Oct. 7) and Hyola50 genotype. Based on the test results, the susceptibility of canola genotypes to temperature, rainfall, and lodging during seed filling period varied between the two years of the experiment. The percentage reduction in seed yield for the Hyola50 and Agamax genotypes was 32% and 40%, respectively. Climatic factors, particularly

temperature and the synchronization of phenological stages with optimal thermal conditions, play a crucial role in determining
1 seed yield, oil content, and fatty acid composition . Furthermore, the selection of heat-tolerant genotypes is essential for maintaining yield stability. Keywords: Canola, Genotype, Heat stress, Oleic acid, Seed filling. 1. Introduction Climate change resulting from global warming is a worldwide concern [1], and the increase in air temperature has led to reduced yield of many crops [2].
20 It is predicted that by 2050, the average global air temperature will increase by 2 C negatively impacting agricultural production and potentially exposing around 50 million people to the risk of hunger [1]. Canola ranks among the most significant oilseed crops worldwide, and rising temperatures impact its developmental stages, seed yield, and oil composition [3]. The timing of sowing is vital for canola cultivation, as the flowering, siliquing, and seed- filling phases should ideally avoid overlap with late-season heat stress [4, 5]. Optimal sowing date and early flowering ensure that flower and silique initiation stages align with favorable environmental conditions (temperature, radiation, moisture), promoting the fertility and development of silique-forming cells. Conversely, exposure of reproductive stages to heat at the end of the season and hot dry winds leads to reduced silique formation and seed yield [4]. Therefore, selecting an appropriate sowing date based on local climatic characteristics is crucial for achieving maximum canola seed yield [5, 6]. Choosing the appropriate sowing date in each region should ensure optimal environmental conditions throughout the growing season for plants without exposure to environmental stresses [4, 7, 8]. Therefore, it is necessary to evaluate the
27 response of genotypes to environmental conditions [9] and identifying the reaction of genotypes to
changes in sowing dates and determining high-yielding genotypes can have a significant impact on expanding canola cultivation [10]. It has been reported that the quantity and quality of canola genotypes in autumn sowing (October) are higher compared to winter planting (January) [11]. Research results in rapeseed have shown that the number of flowers transformed into siliques is a determining factor for seed yield [3, 12] and the production of assimilates during
16 the flowering period determines the number of seeds per silique, while seed weight depends on the
continuity of photosynthesis and assimilate production during the post-flowering period until maturity [12, 13]. Considering that rapeseed reproductive organs are negatively affected by temperatures above 32 C, observing the sowing date plays a crucial role in silique development and seed setting [3]. The temperature during the seed-filling phase affects both the quantity of oil and the composition of fatty acids, with higher temperatures during this time resulting in reduced seed oil content [3]. Environmental stresses, particularly elevated temperatures during seed filling, hinder the production of substrates necessary for triacylglycerol synthesis [3] and diminish fatty acid biosynthesis activities, leading to lower oil content in seeds [14]. An increase in temperature from 19 to 24 C causes changes in canola fatty acids content [15], with the impact of high temperatures between flowering and seed

filling stages on reducing seed oil content and fatty acid changes being more pronounced in sensitive canola genotypes [16]. The role of gibberellin signaling in activating genes involved in fatty acid metabolism and oxalate glycolysis pathway has also been reported [16]. At a certain stage of development, canola seeds contain chlorophyll and feature a thylakoid structure. Light exposure promotes fatty acid biosynthesis in green canola seeds, which may facilitate the production of cofactors and reductants [17, 18]. The transcription factor BnWRI1 regulates the pathways of photosynthesis and fatty acid biosynthesis throughout seed development [19]. The lower chlorophyll a/b ratio in seeds compared to leaves enhances carbon efficiency in oil production [17]. Sucrose is transported from leaves to support seed growth and the synthesis of storage products; however, under high-temperature conditions, oil storage declines, and external sucrose fails

3 to alleviate the adverse effects of heat stress

on oil accumulation. Consequently, the metabolic pathway converting carbohydrates to triacylglycerols is disrupted. Additionally, heat stress conditions lead to decreased expression of certain genes associated with sugar transport in seeds [20]. Photosynthesis in canola embryos significantly boosts oil accumulation efficiency [17, 21], but the activity of photosystem II (PSII) is inhibited by photosynthetic inhibitors, which considerably hampers biomass and oil production in embryos grown under fluorescent light [18]. Ultimately, damage to PSII and suppression of BnWRI1 gene activity are likely key factors contributing to reduced canola oil content under heat stress conditions [20].

5 Canola is one of the most important oilseeds cultivated in many parts of the world, and attention to agricultural factors such as appropriate sowing date and selecting adapted genotypes

26 plays a crucial role in increasing the quantity and quality of this

plant's oil. Many regions in Iran, including warm and dry areas, have faced increased temperatures due to climate change in recent years, posing challenges for farmers on how to escape terminal heat stress as well as choose tolerant and adapted genotypes. Since identifying and planting high-yielding genotypes compatible with climatic conditions can improve both the yield and quality of canola production, the present research aims to identify and introduce the best genotype for different planting dates. 2.

1 Materials and Methods 2.1. Experimental Site The current field experiment was conducted at the Safiabad Agricultural and Natural Resources Research and Education Center

,

18 situated at a latitude of 32°22' N, longitude of 48°32' E, and an elevation of 82 meters above sea level

in Khuzestan province, southwestern Iran. Figure 1a, b illustrate the temperature and precipitation during the experimental period. The

24 soil characteristics are detailed in table 1 . This study was designed as

strip plots within

2 a randomized complete block design , featuring three replications over

two cropping seasons (2017-2019) at the same research center. Seven canola cultivars, both domestic and international (Agamax, Hyola 4815, Hyola 50, Hyola 401, Safi 6, Zabol 9, and

Zabol 13), were assessed across six different sowing dates (
12 September 23, October 7, October 22, November 6, November 21 , and December 6
). The traits of the canola cultivars under evaluation are summarized in table 2. Experimental Design The study was implemented as strip plots within
2 a randomized complete block design with three replications over
two cropping seasons (2017-2018 and 2018-2019). Seven domestic and international canola cultivars (Agamax, Hyola 4815, Hyola 50, Hyola 401, Safi 6, Zabol 9, and Zabol 13) were evaluated across six sowing dates (
12 September 23, October 7, October 22, November 6, November 21 , and December 6
). Experiment Details Prior to sowing, 100
23 kg ha-1 of potassium from potassium sulfate and 70 kg ha-1 of
phosphorus from triple superphosphate were incorporated into the soil. Nitrogen fertilizer amounting to 300 kg ha-1 from urea was applied in three equal portions: one- third before sowing, one-third
5 at stem elongation (BBCH scale: 30), and one-third at flowering (BBCH scale: 61
). For weed control, trifluralin herbicide was applied at 2 l ha- 1 as a pre-sowing soil incorporation, followed by the creation of planting rows 75 cm apart using a furrower. Based on prior research, the planting arrangement consisted of two rows on a 75 cm ridge
10 with a plant density of 60 plants per square meter
(Kalantar Ahmadi 2014a; Kalantar Ahmadi 2014b). Crop management practices and weed control were carried out as required during the growing season. Each experimental plot covered an area of 12 m2. Irrigation was performed using furrows based on
1 70 mm evaporation from a Class A pan
, with 600 cubic meters of water per hectare applied during each irrigation event. Mean and maximum air temperature during silique formation and seed filling period of canola genotypes in different sowing dates shown in table 3. Agronomic Traits During
1 the physiological maturity stage (BBCH scale: 89
), four central rows from each plot were harvested separately over an area of 6 m^2 to assess seed yield. Ten plants were randomly selected to evaluate
1 the number of siliques per plant
.
1 Oil Content and Fatty Acid Composition To assess the oil content
in the seeds, a 5-gram sample from each treatment group was chosen and analyzed using the NMR technique at the Seed Improvement Research Institute laboratory in Karaj. The composition of fatty acids was evaluated through
1 gas chromatography (GS: Agilent Technologies, Santa Clara, CA, US), following the methodology established
2 by Azadmard-Damirchi et al . [22]. Fatty acid methyl esters were produced from the oil samples
. For sample preparation,
1 2 ml of 0.01 M NaOH in methanol was combined with an oil sample dissolved in 0.5 ml of hexane , and this mixture was incubated in a water bath at 60 °C for 10
minutes. Afterward, 20% BF3 in methanol was introduced, and the sample remained

2 in the water bath at 60 °C for
another 10 minutes. Once cooled under running water,
1 2 ml of sodium chloride (20% w/v) and 1 ml of hexane were added to the
mixture. This blend was thoroughly mixed, and then centrifugation was used
22 to separate the hexane layer containing the fatty acid methyl esters
. The analysis of these esters was performed
7 via GC as detailed by Azadmard-Damirchi and Dutta
[23]. Glucosinolates were measured
2 using a spectrophotometer equipped with a CP-Sil 88 capillary column
that is
19 50-m long , has an internal diameter of 0.25 mm, and features a static thickness of 0 .2 µm
[24].
1 Statistical Analysis After conducting Bartlett's test
in order to supply the homogeneity
6 of the test variance in each year, the combined analysis of variance (ANOVA) was conducted using SAS software (version 9.2). The least significant difference (LSD) was used to determine the comparison of means at $p < 0.05$. Excell software was used to draw the graphs. The regression relationship (Stepwise method) between seed yield with phenological characteristics (Flowering initiation, flowering duration period, growth period duration) and temperature (Max, mean and min) was calculated by SPSS software. Cluster analysis was done by method of UPGMA (Euclidean) using Past software (Version 4.03). Results Based on
1 combined analysis of variance results, the simple effect of year
(except stearic acid), sowing date and genotype
5 were significant on all studied traits. The interaction of year
× sowing date was significant on start flowering, growth duration period, seed yield,
7 palmitic acid , stearic acid, linoleic acid, linolenic acid and erucic acid. The interaction of sowing date × genotype, except for oil content, has a significant effect on other studied traits. Amount of growth duration period, seed yield, stearic acid, linolenic acid
1 and erucic acid were impacted by the three-way interaction of year
× sowing date × genotype (Table 4). Flowering Initiation The studied genotypes had various initiate flowering on different sowing dates. Number of days for genotypes to initiate flowering was affected by sowing date. The longest time between sowing and initiation of flowering (86 days after sowing)
3 was observed on the fourth sowing date (Nov. 6) and
Zabol9 and Hyola50 genotypes
11 in the first year (Table 5). In the second year of the experiment, the
longest time interval between sowing and initiation of flowering was also allocated to the fourth sowing date and Zabol9 (91 days after sowing) and Hyola50 (90 days after sowing) genotypes, but this time interval increased compared to the first year (Table 5). The shortest time between sowing and initiation of flowering was observed on
17 the second sowing date (Oct. 7) and Hyola4815 genotype in the first

(45 days after sowing) and second (39 days after sowing) year (Table 5). Flowering duration period Mean comparison between treatments showed that the longest flowering duration period appointed to the first sowing date (Sep. 23) and Zabol9 as well as Zabol13 genotypes in the first (75 days) and second year (103 days). The minimum flowering duration period observed on the last sowing date and Sai6 genotype in the first (19 days) and second year (26 days) of the experiment (Table 5). Growth duration period In
2 the first year, mean comparison of interaction sowing date × genotype revealed that
the maximum growth duration (208 days) observed
3 the first sowing date (Sep. 23) and Hyola50 genotype. The minimum ones
(127 days) appointed to on
3 the last sowing date (Dec . 6) and
Hyola4815 genotype (Table 5). In the second year, Agamax genotype with 222 days on the first sowing date (Sep. 23) and Hyola4815 with 130 days
3 on the last sowing date (Dec . 6) indicated the
highest and the lowest of growth duration, respectively (Table 5). Seed yield In the first year, Agamax genotype achieved
3 the highest seed yield (3357 kg ha-1
) on Oct 7th, but in the following year the peak seed yield (2888.9
4 kg ha-1) belonged to the second sowing date
(Oct. 7) and Hyola50 genotype (Table 5). The lowest seed yield were noted for Agamax
(1100
16 kg ha-1) and Zabol9 (385 kg ha-1) genotypes
when they were planted on Dec. 7
2 in the first and second year, respectively (Table 5). The analysis of regression between seed yield
and temperature along with phenological traits indicated that growth period duration and flowering initiation had positive effect, but maximum temperature had negative effect on seed yield in the first year. Seed yield_(year 1)=541.99+22.42 Growth period duration-123.43 Max temperature+15.93 Flowering initiation There was a positive relationship between growth period duration and seed yield, as well as a negative relationship between seed yield and maximum temperature observed in the second year. Seed yield_(year 2)=141.94+13.98 Growth period duration-58.81 Max temperature Oil content The average oil content varied significantly over the two years; specifically, it was
21 higher in the first year at 41.11% compared to 40. 6% in the second
year (data not presented). The delay in sowing date
31 resulted in a reduction of oil content. The results revealed that the
maximum (42.05%) and the minimum (39.45%) oil content belonged to the first (Sep. 23) and the last (Dec. 6) sowing date during two years of the experiment (Fig. 2a). Between studied genotypes, Hyola50 and Hyola4815 produced the highest (43.04%) and the lowest (38.81%) oil content, respectively (Fig. 2b). Palmitic acid Delaying sowing dates
5 led to a decrease in oil content . A comparison of
means for the

2 interaction between sowing date and genotype revealed that Safi6 produced the highest palmitic acid
content (5.93%) on the first sowing date (Sep. 23), while Zabol13 yielded the lowest amount (3.6%)
3 on the final sowing date (Dec . 6) during the
first experimental year (Table 5). The results showed that the maximum palmitic acid (8.05%) belonged to the second sowing date (Oct. 7) and Hyola401 genotype, while Zabol13 genotype produce the minimum palmitic acid (3.42%) on the last sowing date in the second year (Table5). Stearic acid The
6 means comparison of two-way interaction of sowing date and genotype
illustrated that
3 the maximum amount of stearic acid
(6.48%) was appointed
3 to the sowing date on Nov . 21 and
Hyola401 genotype
11 in the first year. In contrast, during the second year
, Hyola401 again showed high levels of stearic acid at 5.61% on Oct. 7 (Table 6). The lowest stearic acid (3.12%) was found in Agamax and Zabol13 genotypes in the first sowing date (Sep. 23)
15 in the first year and in the second year the
minimum ones appointed to Zabol13 in sowing date Nov. 21 (Table 6). Oleic acid Based on mean comparison results, the canola genotypes responded differently to sowing date regarding oleic acid, so that that
28 the highest amount of oleic acid observed in Hyola50 in the
last sowing date (Dec. 6) in the first (63.38%) and second year (60.89%). Zabol9 genotype had the lowest oleic acid (49.6%) on the first sowing date (Sep. 23)
15 in the first year , while in the second year the
minimum ones (50.02%)
4 belonged to the first sowing date (Sep . 23) and
Zabol13 genotype (Table 6). Linoleic acid A notable difference was found in how the studied genotypes responded to changes in sowing dates regarding linoleic acid content. The average comparison indicated that in both the first (27.26%) and second (26.17%) years, the Hyola50 genotype produced the highest levels of linoleic acid on the initial sowing date (Table 6). Conversely,
3 on the last sowing date (Dec . 6), the lowest linoleic acid
levels were recorded for the Zabol13 genotype in both the first (15.6%) and second years (14.62%) (Table 6). 3.10. Linolenic acid The maximum amount of linolenic acid (5.92%) belonged Hyola401 in the second sowing date (Oct. 7) in the first year, while Safi6 produced the maximum (6.81%) ones when planted on Sep. 23 in the second year (Table 6). The minimum values were obtained from Zabol13 genotype with averages of 2.15 and 2.25% when planted on Dec.
1 6 in the first and second year, respectively (Table

6). 3.11. Erucic acid When compared treatments in the first year, Safi6 genotype produced the highest erucic acid (0.418%) on the second sowing date (Oct. 7), as well as that Hyola50 and Hyola4815 produced the lowsest erucic acid by 0.015 and 0.016%, respectively
2 on the last sowing date (Dec . 6) (Table 6). The average erucic acid of
Safi6 on Oct. 7 was 0.363% , while the amount of this trait for Agamax genotype grown
3 on the last sowing date (Dec . 6) was the
minimum amout (0.036%)
3 in the second year (Table 6). 3
.12. Glucosinolate During two years, canola genotypes significantly differed in the amount of glucosinolate in sowing dates. The studied genotypes had a significant difference in the amount of glucosinolate in sowing date during two years of the experiment. In the first year, based on two-way interaction of sowing date × genotype, the maximum amount of glucosinolate detected to Safi6 (8.44
29 µmol g-1) and Hyola4815 (8.41 µmol g-1) on the
last sowing date (Dec. 6), while Zabol13 genotype produced the minimum amount (2.61 µmol g-1) of this trait on Sep. 23 (Table 6). In the second year, the highest (8.35 µmol g-1) glucosinolate content appointed to the last sowing date (Dec. 6) and Safi6 genotype, while Agamax genotype with an average of 2.7 µmol g-1 showed the lowest ones (Table 6). 4. Discussion The variations observed in the studied traits can be linked to differences in climatic conditions (Temperature, humidity, rainfall, etc), and the interactions among these factors throughout the growth period (Fig. 1a, b), particularly temperature during flowering and seed filling. This study examined temperature and sowing date as key environmental factors influencing plant adaptation to their surroundings (Table 3). Notably, differences
3 in temperature due to changes in sowing date
and the length of phenological stages are significant. The timing of flowering initiation and the duration of flowering varied among genotypes in different sowing dates during both years. In the first and second year of the experiment, Zabol9 and Hyola50 genotypes had the longest time between sowing and flowering initiation on the fourth sowing date (Table 5). The shortest interval between sowing and flowering initiation was observed for the Hyola4815 genotype on the second sowing date; this interval occurred six days earlier
14 in the second year compared to the first year
, highlighting
9 the influence of environmental conditions on genotype responses. In terms of
flowering duration, Zabol9 and Zabol13 genotypes exhibited the longest flowering periods during both years of the study. However, higher temperatures
7 in the first year of the experiment compared to the second year
led to a reduction in the flowering duration period (Fig. 1). These results were consistent with some researches about the effect of environment on rapeseed flowering [25, 26]. The postponement of the sowing date resulted in a shorter flowering duration across all genotypes evaluated. This delay in planting coincided with rising environmental temperatures during the flowering phase, which contributed to a
17 reduction in the length of the flowering period

. Reduction the flowering duration period means that there is less opportunity for the formation of siliques, and this can lead to a decrease in seed yield [27, 4]. Delayed sowing reduced the growth period duration in all genotypes tested. Hyola4815 genotype was the earliest genotype and had the shortest growth period in all sowing dates (Table 5). In general, delayed planting led to a reduction in the growth period, emphasizing the importance of selecting appropriate sowing dates for optimal plant development and seed yield in canola. Khayat et al. [9] stated that with the onset of flowering, the process of photosynthesis decreases significantly, and the plant becomes sensitive to environmental stresses such as high temperatures. Improper sowing dates leads to reduce the growth period duration in canola genotypes, and terminal heat stress reduce seed filling period and transfer of photosynthetic materials to seeds, ultimately reducing seed yield. Flowering is one of the important stage to transition to the reproductive phase, which is controlled by different genes, and under stress conditions, the plant uses different mechanisms to change flowering in order to maintain survival [28]. Environmental conditions, including high temperatures during vegetative and reproductive periods, are determinants of seed yield [4, 29, 30]. The response of the genotypes under investigation to climatic conditions differed between the two years of the experiment. Despite the fact that the siliquing and seed-filling periods of the genotype Hyola4815 did not coincide with high temperatures (Table 3), its seed yield was lower than that of the other genotypes. This can be attributed to the inherently low yield potential of this genotype. Climatic variations between the two years of the experiment led to changes in the yield of canola genotypes, with most genotypes exhibiting lower seed yield in the

3 second year. The reduction in seed yield of the

Hyola50 genotype was less compared to other varieties (Table 5), suggesting that this genotype has greater yield stability under changing weather conditions than the other genotypes. Although temperatures during the siliquing and seed-filling periods were lower

2 in the second year compared to the first year (Table 3

), and higher seed yields were expected, heavy rainfall (104 mm over two consecutive days) in late-March of the second year (Fig. 1), along with strong winds, caused lodging of plants. This led to silique shattering due to temperature fluctuations, seed shedding, and ultimately reduced seed yield. Furthermore, plant lodging stimulated a re-flowering process, which also contributed to the reduction in seed yield. The lodged plants not only failed to return to their original upright position but also initiated new flowering. The newly formed flowers did not positively affect yield; instead, they were damaged by high temperatures and failed to produce seeds. Consequently, assimilates that should have been allocated to seed filling were diverted to support the newly formed flowers, leading to further reductions in seed yield. The negative impact of lodging on seed yield through disruption of the accumulation and distribution of photosynthates and nutrients has been reported [31, 32]. Moreover, the timing of lodging occurrence affects yield loss, with lodging after flowering causing more severe yield reductions [33, 34, 35]. The detrimental effects of lodging on grain yield are more pronounced during post-flowering stages. The results of cluster analysis using the UPGMA method for seed yield across different sowing dates for seven genotypes studied in the first

year indicated that the results could be grouped into three main clusters (Fig. 3). Group A included the first, second, and occasionally third sowing dates. In this group, the genotype Agamax
33 had the highest grain yield (3357.04 kg ha-1
) on the third planting date (Oct. 22), followed by Agamax on the first sowing date (Sept. 23) and Hyola50 on the fourth sowing date, ranking second and third, respectively (Table 5). Group B generally included the third, fourth, and fifth sowing dates, indicating that intermediate sowing dates were represented in this group (Fig. 3). The results showed that genotype Hyola401 had
4 the highest seed yield (2582.52 kg ha-1) on the fourth planting date
(Nov. 6), followed by Zabol9 on the same date and Hyola50 on the Nov. 21 (Table 5). Group C included the sowing dates of Nov. 21 and Dec. 6 (Fig. 3), with genotype Hyola4815 achieving
4 the highest seed yield (1801.11 kg ha-1) on Dec. 6. The
mean seed yield in groups A, B, and C in the first year (Fig. 3) was 3038, 2328, and 1592 kg ha-1, respectively. The average temperatures on the sowing dates of Sep. 23, Oct. 7, and Oct. 22 were 25.53, 25.72, and 25.91 ºC, respectively and from the sowing date of Nov. 6 onwards, the maximum temperature rose above 28 ºC. Therefore, the reason that the first (Sep. 23) and second (Oct. 7) sowing dates were predominantly in group A can be attributed to the lower temperatures during these planting periods throughout the seed filling period. Kalantarahmadi & Daneshian [4] also stated that for each unit increase in temperature above 28 ºC during seed filling period, seed yield decreases by 340 kg ha-1. Cluster analysis in the second year showed that the obtained results could be grouped into two main clusters (Fig. 4). Group A included the first, second, third and occasionally forth sowing dates. In this group, Hyola50 in the second sowing date (Oct. 7) had the maximum seed yield (2781.48 kg ha-1), followed by Hyola50 and Zabol9 on sowing dates Sep. 23 and Oct. 7, respectively (Table 5). Group B encompassed
30 sowing dates of Nov. 6, Nov. 21 and Dec. 6
. The mean seed yield in Groups A and B in the second year (Fig. 4) were recorded at 1903 and 849 kg ha-1, respectively. Despite experiencing heavy rainfall and lodging that reduced seed yields in the second year of the study, early sowing dates within Group A still produced higher yields. Based on the results from the two years of testing, it can be stated that considering the climatic conditions of the region and the tested genotypes, delayed sowing dates (after Nov. 6) are not recommended. Comparison of average sowing dates in two years of study showed that delaying sowing date reducing the silique period, and seed filling period
5 led to a decrease in oil content
(Fig. 2a). Oil content is affected by temperature during the seed filling stage; increased temperatures during this time lead to a decrease in seed oil content. Environmental stresses such as elevated temperatures during seed filling hinder the production of precursors necessary for triacylglycerol synthesis [3] and fatty acid biosynthesis activities [36], ultimately

resulting in lower seed oil content [14]. Heat stress also reduces oil content due to disruptions in oil biosynthesis [36]. Climatic conditions and sowing date significantly affect oil content, as plant productivity depends on aligning growth stages with environmental conditions. The difference in seed oil content in the evaluated genotypes can be attributed to their genetic differences and the effect of environmental factors on the amount of oil is less compared to other traits [37, 38]. Photosynthesis by green seeds and siliques induces the formation of essential components of the fatty acid biosynthesis pathway [20] and control of this pathway is carried out by the transcription factor BnWRI1 [19]. Heat stress also
25 reduces the amount of oil by inhibiting photosynthesis and reducing the expression of many genes [20]. Palmitic acid and stearic acid are recognized as two primary unsaturated fatty acids [52]. Climatic conditions impacted palmitic acid levels, which were
14 higher in the second year (5.54%) compared to the first year
(4.7%), while stearic acid levels remained consistent between years one (4.44%) and two (4.46%), showing no significant difference (data not shown). Although some research indicates an increase in saturated fatty acids under high-temperature conditions, our findings revealed that elevated temperatures during seed filling
5 in the first year led to a decrease in
palmitic acid compared to those observed in the second year (data not shown), with varying responses among genotypes. Genotype Hyola4815, being one of the earliest genotypes with a shorter growth period, had high palmitic acid content throughout all sowing dates compared to the late-maturing genotype Hyola50, indicating the significant role of genetic traits in determining palmitic acid amount. The higher amount of stearic acid in Hyola401 in the first and second year of the experiment indicates the genetic characteristics of this genotype regarding the high level of stearic acid, but considering the fact that it has changed in the two years of the experiment with different sowing dates, it indicates the effect of environmental factors on the amount of stearic acid. While genetic traits influence oil quantity and fatty acid composition, temperature conditions during seed filling also play a crucial role [3, 4]. Enzymes involved in oil biosynthesis are sensitive to high temperatures; thus, an increase in temperature can lead to reduced oil content [39]. Some studies have found no impact on unsaturated fatty acid levels due to temperature variations [3], while others report an increase in oleic acid alongside a decrease in linoleic acid [40]. The degree and timing of temperature effects on fatty acids depend on both intensity and timing of heat exposure; current results align with other studies indicating that delayed sowing dates can positively affect oleic acid levels while reducing linoleic acid during seed filling (Table 6) [4, 40]. The rise in oleic acid at elevated temperatures may stem from limitations on converting oleic acid into linoleic acid as a precursor. This increase could be linked to restricted conversion processes under high-temperature conditions [41]. The enzyme oleate desaturase is responsible for converting oleic acid into linoleic acid through cytosolic desaturation; variations in its activity may explain
2 shifts in the oleic/linoleic ratio under drought and heat
stress conditions [42]. Linoleic acid is produced from oleic acid desaturation [43], with each

step from oleic to linoleic being potentially influenced by both temperature duration and genetic factors [44]. Furthermore, different genotypes exhibit varying sensitivities within their fatty acids' biosynthesis metabolic pathways when exposed to temperature changes [44]. Irrigation can also mitigate heat stress and influence the activity of the enzyme oleate desaturase, thereby affecting fatty acid levels [45]. Elevated temperatures during the seed filling stage result in a rise in oleic acid levels while causing a decline in linolenic acid. Moreover, the duration of high temperatures has a greater impact on fatty acid composition compared to the timing of their occurrence [40]. Although the responses of the tested genotypes regarding changes in oleic and linolenic acids varied across different sowing dates, generally, in delayed sowings where temperatures were higher during the seed filling period, oleic acid increased while linolenic acid decreased. Research has also indicated that high temperatures positively influence the increase of oleic acid more significantly than that of linolenic acid [46]. Fluctuations in temperature during this period lead to alterations in fatty acid composition [47, 48], with oleic and linolenic acids in canola being particularly responsive to these temperature variations [49]. The final amount of linolenic acid is determined by the desaturation rate of C18:1 to produce C18:2, followed by the conversion of C18:2 into C18:3 [46]. Variations in the fatty acid profiles among rapeseed cultivars can be attributed to their genetic diversity, and differences observed among the genotypes tested align with findings from other studies [50, 51, 52]. Erucic acid is a significant fatty acid concerning its effects on

2 human health, and its concentration is affected by both genetic factors and environmental conditions

[54, 4]. Delay in sowing date was found to reduce erucic acid levels in the current experiment, with genotype responses differing, indicating environmental factors influence on erucic acid. These results align with Gharechaei et al. [45] findings on reduced erucic acid in late plantings. An increase in glucosinolate content within seeds negatively impacts the quality and nutritional value of rapeseed meal [55], which is also influenced by genetic and environmental variables [56]. Unlike erucic acid, glucosinolate levels increase with delayed sowing dates, consistent with findings by other researches [4, 54]. It seems that when the seeds are in the filling stage and the plant is faced with heat stress [4], the amount of glucosinolates in vegetative tissues and siliques then remobilized to seeds and finally its amount increases in the seed [57]. Halting mineral and metabolite transfer to seeds also stops glucosinolate transport [58]. Approximately 78 loci related to glucosinolates have been identified, with 36 genes involved in glucosinolates biosynthesis. The key gene is BnaA03g40190D, determining high leaf glucosinolates and low seed glucosinolates, contributing to increased resistance of rapeseed genotypes against pests and diseases [59].

2 In this study, the levels of erucic acid (below 2%) and glucosinolate (under 30 µmol g1

) were found to be within acceptable limits [7]. 5. Conclusion Achieving optimal seed yield in canola necessitates the plant's adaptation to key environmental parameters, including temperature, humidity, and both the spatial and temporal distribution of rainfall. Hence, the timing of sowing, in conjunction with other environmental factors, represents a critical

agronomic determinant influencing the plant's adaptation to its surroundings, ultimately shaping both the
32 yield and quality of canola production. This study underscores the pivotal role of temperature in modulating seed yield and the composition of fatty acids. Postponing the sowing date (from Sep. 23 to Dec. 6) and elevating the mean temperature from 17.98 °C to 23.17 °
8 C in the first year, and from 15.83 ° C to 20.95 ° C in the second year, during the silique formation and seed filling stages, induced alterations in both seed yield and fatty acid composition across the examined genotypes. The Hyola50 genotype exhibited a comparatively lower reduction in seed yield than the other assessed genotypes. The differences in the responses of the studied genotypes indicate genetic diversity in reaction to temperature and environmental conditions. Therefore, heat tolerance is a trait that should be considered in breeding programs due to climate change. CRediT authorship contribution statement Seyed Ahmad Kalantar Ahmadi: Conceptualization (equal); Investigation, Software, Formal analysis, Writing- Original draft preparation. Mohsen Sarhangi: Re-Writing, Software. Funding statement There was no source of funding for the manuscript from any public, private, or non- profit funding bodies was allocated to this study.
34 Data availability statement Data is not available for the
statement. Declaration of
13 competing interest The authors declare that they have no conflict of interest. Additional information No additional information is available for this paper. Acknowledgments
Thanks to both Agricultural Research, Education and Extension Organization (AREEO) as well as Safiabad Agricultural and Natural Resources Research and Education Center.

sources:

1

162 words / 3% - Crossref

Seyed Ahmad Kalantar Ahmadi, Hamed Eyni-Nargeseh. "Foliar Application of Growth Regulators Mitigates Harmful Effects of Drought Stress and Improves Seed Yield and Oil Quality of Rapeseed (Brassica napusL.)", Gesunde Pflanzen, 2023

2

134 words / 2% - from 15-Apr-2023 12:00AM

link.springer.com

3

92 words / 2% - Crossref

Seyedahmad Kalantarahmadi, Jahanfar Daneshian. "Improving of Canola (Brassica napus L.) Yield and Oil Quality by Foliar Application of Micro-nutrients Under High-Temperature Stress", Journal of Soil Science and Plant Nutrition, 2023

4

72 words / 1% - from 25-Mar-2024 12:00AM

agrobreedjournal.ir

5

57 words / 1% - Crossref
S. A. Kalantar Ahmadi, J. Daneshian. "Improving Canola (Brassica napus L.) Seed Yield through Micronutrients Sprays: Effects on Enzymatic and Non-Enzymatic Defense under Different Irrigation Regimes", Russian Journal of Plant Physiology, 2024
6
46 words / 1% - Crossref
Saber Saif Amiri, Mehrdad Yarnia, Bahram Mirshekari, Farhad Farahvash, Varahram Rashidi. "Restricted Irrigation Regimes and Rapeseed High-Yielding Genotypes Can Be Applied to Cope With the Water Shortage Crisis and More Stable Oil Production", Journal of Crop Health, 2024
7
29 words / < 1% match - Crossref
Ghorban Khodabin, Zeinolabedin Tahmasebi-Sarvestani, Amir Hossein Shirani Rad, Seyed Ali Mohammad Modarres-Sanavy et al. "Effect of Late-Season Drought Stress and Foliar Application of ZnSO4 and MnSO4 on the Yield and Some Oil Characteristics of Rapeseed Cultivars", Journal of Soil Science and Plant Nutrition, 2021
8
11 words / < 1% match - Internet
Fábio Marcon Alfieri, Caren da Silva Dias, Daniela Mitiyo Odagiri Utiyama, Denise Vianna Machado Ayres et al. "The Immediate Effect of Exercising in a Virtual Reality Treadmill (C-Mill) on Skin Temperature of a Man with Lower Limb Amputation", Case Reports in Vascular Medicine
9
8 words / < 1% match - Internet
A.S. Lyzhin, I.V. Luk’yanchuk. "Study of a genetic collection of strawberry (Fragaria L.)for resistance to powdery mildew", Vavilov Journal of Genetics and Breeding
10
8 words / < 1% match - Internet
Hedayat Karimzadeh, Azam Borzouei, Behnam Naserian, Seyyed Ali Tabatabaee, Mohammad Reza Rahemi. "Investigating the response mechanisms of bread wheat mutants to salt stress", Scientific Reports
11
22 words / < 1% match - Crossref
Juozas Lanauskas, Darius Kviklys, Mindaugas Liaudanskas, Valdimaras Janulis, Nobertas Uselis, Jonas Viškelis, Pranas Viškelis. "Lower nitrogen nutrition determines higher phenolic content of organic apples Juozas Lanauskas1, Darius Kviklys1, Mindaugas Liaudanskas2, Valdimaras", Horticultural Science, 2017
12
22 words / < 1% match - Crossref
Kasilingam Lingaraja, Justin Paul, Murugesan Selvam. "Indian culture, lunar phases and stock market returns", International Journal of Indian Culture and Business Management, 2019
13
20 words / < 1% match - Internet from 08-Dec-2022 12:00AM
core.ac.uk

14
19 words / < 1% match - Internet
Ton Aybegün. "Response of cowpea (vigna unguiculata l.) to organic and chemical fertilizer applications on grain yield and yield components", BIO Web of Conferences, 2024
15
19 words / < 1% match - Internet from 15-Feb-2017 12:00AM
www.mdpi.com
16
18 words / < 1% match - Internet
rfan Öztürk. "Flag Leaf of Bread Wheat (Triticum aestivum L.) Genotypes and Relation with Yield Component under Rainfed Conditions", International Journal of Innovative Approaches in Agricultural Research, 2023
17
16 words / < 1% match - Crossref
Mahdi Shamsi. "Study of the Effects of Planting Date on the Phonological and Morphological Features, the Seed Yield, and the Components of the Yield of Oilseed Rape", International Journal of Biology, 12/29/2011
18
16 words / < 1% match - Crossref
Shiv Bhushan Verma, Chitranjan Kumar, Raghvendra Pratap Narayan. "Enhancing Soil Health through Organic Inputs to Sustain Tomato Production under Saline Conditions", BIO Web of Conferences, 2024
19
16 words / < 1% match - from 24-Feb-2024 12:00AM
www2.mdpi.com
20
12 words / < 1% match - Crossref
Abdulaziz I. Almulhim, Khalid Mohammed Almatar. "Chapter 38 Level of Awareness and Knowledge Regarding Climate Change Among the People of Dammam, Saudi Arabia", Springer Science and Business Media LLC, 2023
21
11 words / < 1% match - Internet
Darby, Heather, Ziegler, Sara, Calderwood, Lily, Cummings, Erica, Gupta, Abha, Post, Julian. "Summer Annual Forage Mixtures Trial", UVM ScholarWorks, 2015
22
11 words / < 1% match - Internet from 10-Aug-2012 12:00AM
www.waset.org
23
10 words / < 1% match - Internet
Takahashi, Mário, Fidalski, Jonez. "Soil tillage to produce cassava roots with less physical limitations in pasture succession", Universidade Estadual do Oeste do Parana - UNIOESTE, 2019
24
9 words / < 1% match - Crossref
A. WOLNA-MARUWKA, A. NIEWIADOMSKA, A. GRZYB, T. PIECHOTA, M. SZCZECH, P.

RYCHLIK. "THE INFLUENCE OF ORGANIC FERTILISER PROTOTYPES WITH THE TRICHODERMA ON THE SANITARY AND ENZYMATIC CONDITIONS OF SOIL AND THE YIELD OF SPINACH (SPINACIA OLERACEA L.)", Applied Ecology and Environmental Research, 2020
25
9 words / < 1% match - Crossref
Gokul Ram Nishad, Ashwani Kumar Sharma, Dakeshwar Kumar Verma. "Chapter 5 Carbon allotropes: mechanism of corrosion prevention and control", Walter de Gruyter GmbH, 2022
26
9 words / < 1% match - Crossref
Hee Jin, So-Yeon Park, Ji Eun Lee, Hangyeol Park, Michaela Jeong, Hyukjin Lee, Jaeho Cho, Yun-Sil Lee. "GTSE1-driven ZEB1 stabilization promotes pulmonary fibrosis through the epithelial-to-mesenchymal transition", Molecular Therapy, 2024
27
9 words / < 1% match - Publications
Robert M. Goodman. "Encyclopedia of Plant and Crop Science", CRC Press, 2004
28
8 words / < 1% match - Crossref
Ashkan Ashkiani, Saeed Sayfzadeh, Amir Hossein Shirani Rad, Alireza Valadabadi, Esmaeil Hadidi Masouleh. "Effects of foliar zinc application on yield and oil quality of rapeseed genotypes under drought stress", Journal of Plant Nutrition, 2020
29
8 words / < 1% match - Crossref
Bhandari, Shiva, and Jung-Ho Kwak. "Chemical Composition and Antioxidant Activity in Different Tissues of Brassica Vegetables", Molecules, 2015.
30
8 words / < 1% match - Crossref
C.F. Shaykewich, P.R. Bullock. "Modeling Canola Phenology", American Society of Agronomy, 2018
31
8 words / < 1% match - Crossref
Jun Lu, Guangliang Li, Kuifeng He, Weiqin Jiang et al. "Luteolin exerts a marked antitumor effect in cMet-overexpressing patient-derived tumor xenograft models of gastric cancer", Journal of Translational Medicine, 2015
32
8 words / < 1% match - Internet from 22-Sep-2022 12:00AM
downloads.hindawi.com
33
8 words / < 1% match - Internet
Ayalew, Abay, Beyene, Sheleme, Walley, Fran. "Response of Haricot Bean Varieties to Different Levels of Zinc Application in Selected Areas of Ethiopia", Food Science and Quality Management, 2015
34
6 words / < 1% match - Crossref
Chenchen Xu, Shou Zhang, Baozhong Sun, Peng Xie, Xiaochang Liu, Lan Chang, Fushan

Lu, Songshan Zhang. "Dietary Supplementation with Microalgae (Schizochytrium sp.) Improves the Antioxidant Status, Fatty Acids Profiles and Volatile Compounds of Beef", Animals, 2021
